# Highly hydroxylated hafnium clusters are accessible to high resolution EUV photoresists under small energy doses†

**DOI:** 10.1039/d3na00508a

**Published:** 2023-11-10

**Authors:** Yu-Fang Tseng, Pin-Chia Liao, Po-Hsiung Chen, Tsai-Sheng Gau, Burn-Jeng Lin, Po-Wen Chiu, Jui-Hsiung Liu

**Affiliations:** a Department of Chemistry, National Tsing Hua University Hsinchu 30013 Taiwan rsliu@mx.nthu.edu.tw; b TSMC-NTHU Joint Research Center, National Tsing Hua University Hsinchu 30013 Taiwan; c College of Semiconductor Research, National Tsing Hua University Hsinchu 30013 Taiwan

## Abstract

This work reports the success in accessing high-resolution negative-tone EUV photoresists without radical chain growth in the aggregation mechanism. The synthesis of a highly hydroxylated Hf_6_O_4_(OH)_8_(RCO_2_)_8_ cluster 3 (R = *s*-butyl or *s*-Bu) is described; its EUV performance enables high resolution patterns HP = 18 nm under only 30 mJ cm^−2^. This photoresist also achieves high resolution patterns for e-beam lithography. Our new photoresist design to increase hydroxide substitutions of carboxylate ligands in the Hf_6_O_4_(OH)_4_(RCO_2_)_12_ clusters improves the EUV resolution and also greatly reduces EUV doses. Mechanistic analysis indicates that EUV light not only enables photolytic decomposition of carboxylate ligands, but also enhances the Hf-OH dehydration. One additional advantage of cluster 3 is a very small loss of film thickness (*ca.* 13%) after the EUV pattern development.

## Abstract 2

This work reports the success in accessing high-resolution negative-tone EUV photoresists without radical chain growth in the aggregation mechanism.

## Graphical abstract

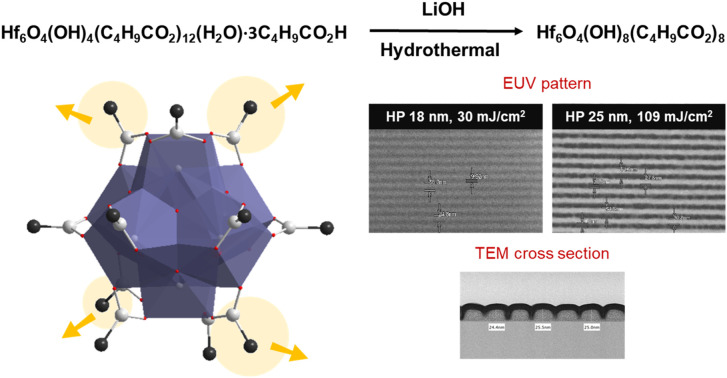

## Introduction

**Scheme 1:**
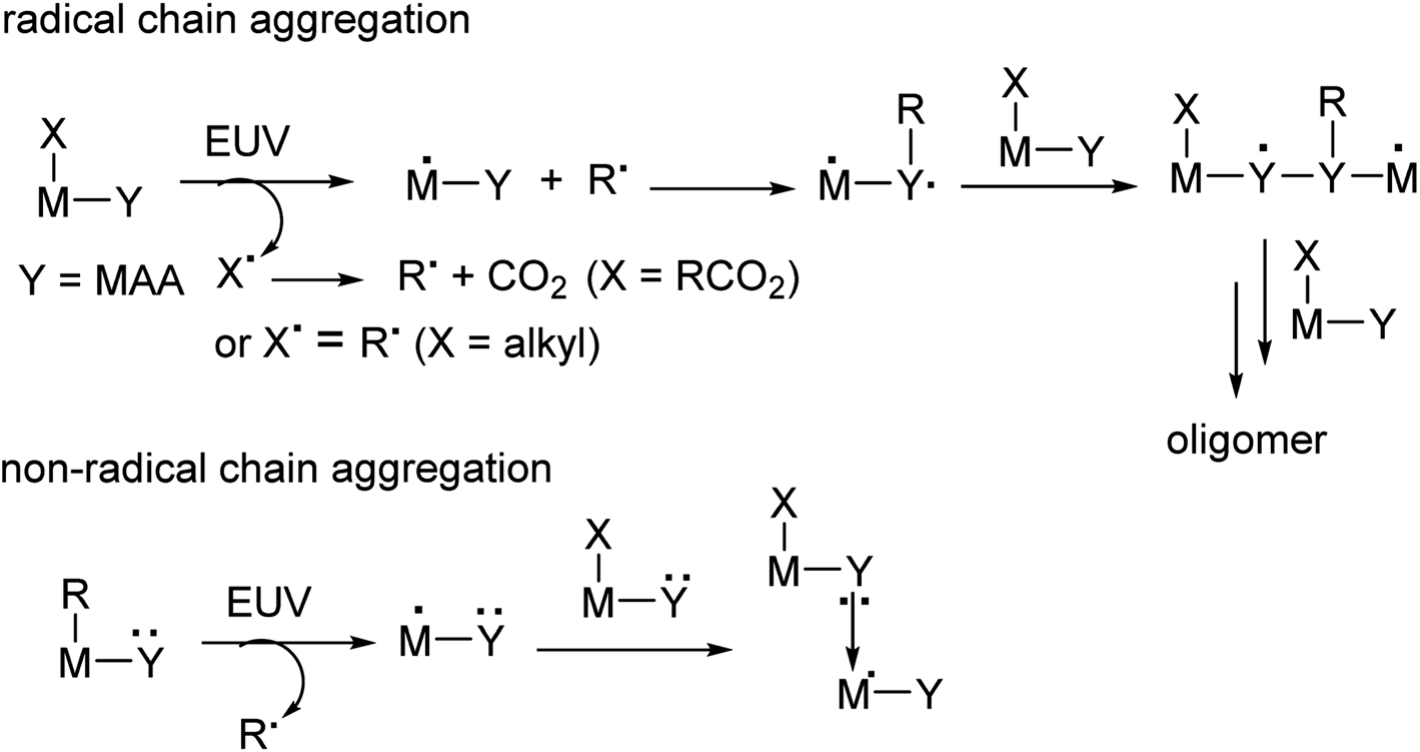
Types of molecular aggregations for negative-tone photoresists.

Metal-based photoresists have gained growing interest in EUV lithography because of their strong absorption ability.^1–5^ High resolution patterns with half-pitches (HP < 20 nm) are required to produce sub-7 nm node IC chips. Many EUV photoresists typically employ metal clusters or metal nanoparticles as negative-tone photoresists; these materials have high metal density to utilize EUV very efficiently. As the cost of EUV light is very high because only 3–4% of the light is used for lithography, development of new EUV photoresists using low doses (<50 mJ cm^−2^) is given prior consideration. Embedment of a radical acceptor such as methylacrylate (MAA) onto metal clusters or nanoparticles is a current approach to reduce EUV doses.^6–16^ A general protocol is depicted in Scheme 1, in which carbon radicals (R˙) attack at a MAA ligand to induce radical chain growth for molecular aggregation. Nevertheless, very few photoresists can reach high resolution patterns with HP <20 nm.^17,18^ Radical chain growth on a MAA ligand may also occur rapidly between exposed and unexposed photoresists due to small kinetic barriers. Negative-tone photoresists are probably a viable route to avoid radical chain growth, although there are no successful examples. (*n*-BuSn)_12_O_14_(OH)_6_X_2_ (ref. 19–22) clusters represent special photoresists, in which molecular aggregations do not rely on radical chain growth. With EUV light, the *n*-BuSn bonds are readily cleaved to create empty sites that are coordinated by oxide or hydroxide ligands. Although such tin oxide photoresists achieve a high resolution pattern with HP = 18 nm (X = acetate), the EUV doses are very high (>200 mJ cm^−2^). We sought highly hydroxylated clusters to reduce EUV energy because hydroxide ligands (M−Y = M–OH) are potent nucleophiles that can facilitate a molecular aggregation process.

**Scheme 2:**
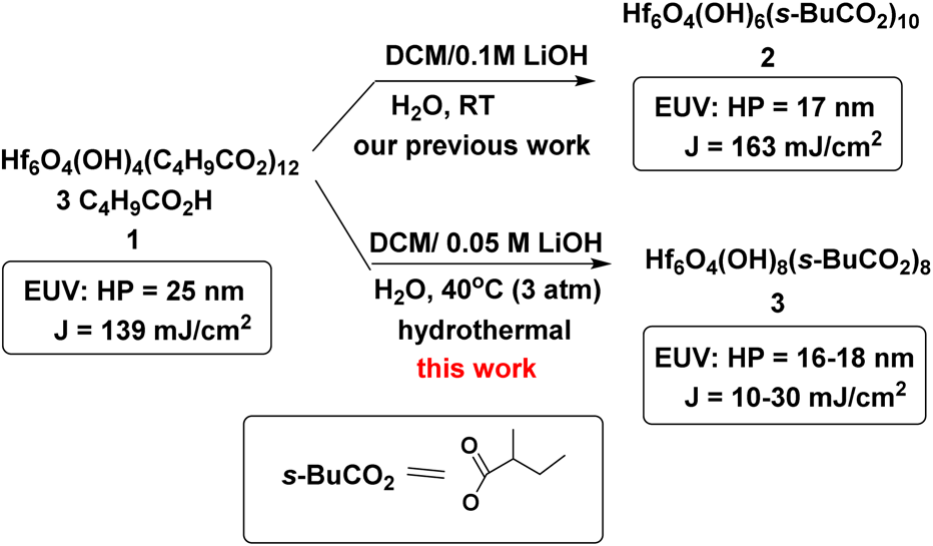
Hydroxide ligand effects of hafnium oxide clusters.

Hafnium oxide photoresists are the best-studied clusters for the development of negative-tone EUV photoresists. Hf_6_O_4_(OH)_4_(RCO_2_)_12_ clusters^6,13,23^ were selected as the platform (see Scheme 2). Our desired EUV photoresists are to avoid a radical chain aggregation; a MAA ligand is now replaced with 2-methylbutyrate. The synthesis involves multiple hydroxide (OH^−^) substitutions of the carboxylated ligands as in Hf_6_O_4_(OH)_4_(RCO_2_)_12_ clusters. The molecular sizes of new hafnium clusters become smaller, thus facilitating molecular aggregation. As mentioned before, the potent nucleophilicity of the Hf-OH ligand is also favorable to form dimeric Hf-O-Hf species (see Scheme 1). In our recent work,^23^ the EUV pattern of Hf_6_O_4_(OH)_4_(*s*-BuCO_2_)_12_ cluster 1 was resolved to HP = 25 nm, with a dose *J* = 139 mJ cm^−2^ (see Scheme 2). As a comparison, its doubly hydroxylated cluster Hf_6_O_4_(OH)_6_(*s*-BuCO_2_)_10_2 has an EUV pattern resolving to HP = 17 nm, but the dose is still very high (*J* = 163 mJ cm^−2^).^23^ The resolution improvement is attributed to a non-radical chain process, but a high EUV dose for cluster 2 is beyond our photoresist design. We believe that the size of cluster 2 is not sufficiently small. This work reports the synthesis of highly hydroxylated Hf_6_O_4_(OH)_8_(*s*-BuCO_2_)_8_ cluster 3; importantly, the cluster is accessible to a resolution pattern with HP = 18 nm with a small EUV dose (*J* = 30 mJ cm^−2^). The energy-saving mechanism has been elucidated to involve two EUV-activated aggregations, including (i) a Hf-OH dehydration and (ii) a photolytic decarboxylation. Apart from EUV performance, this new material can be resolved into HP = 20 nm using an e-beam as the energy source. Unlike common negative-tone EUV photoresists, cluster 3 has a small loss (*ca.* 13%) of film thickness for EUV lithography after pattern development.^24^

## Results and discussion

**Fig. 1:**
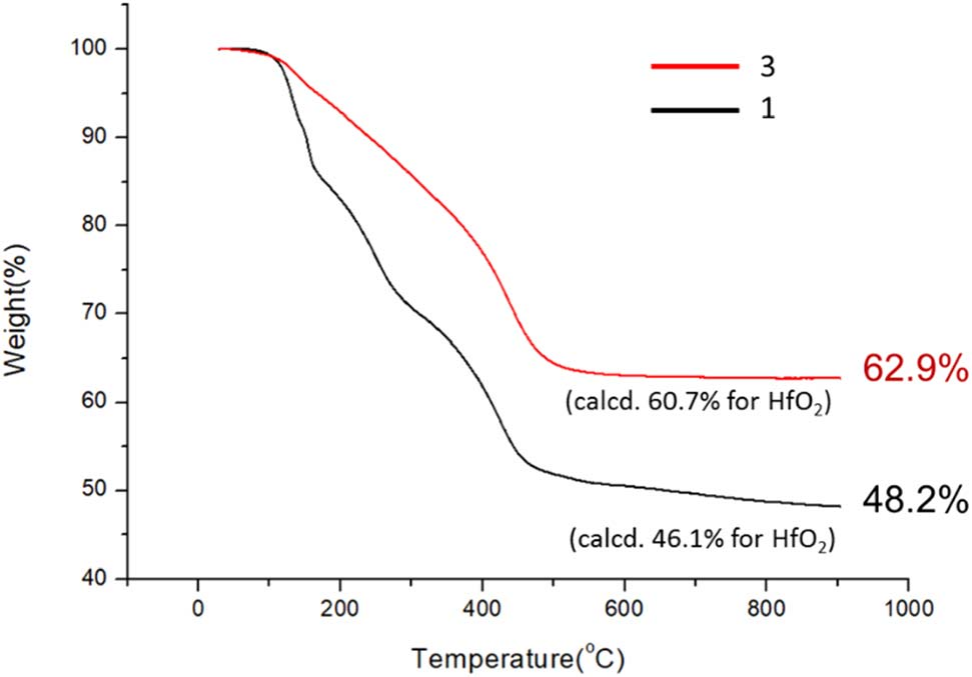
TGA analysis for cluster 3.

**Fig. 2:**
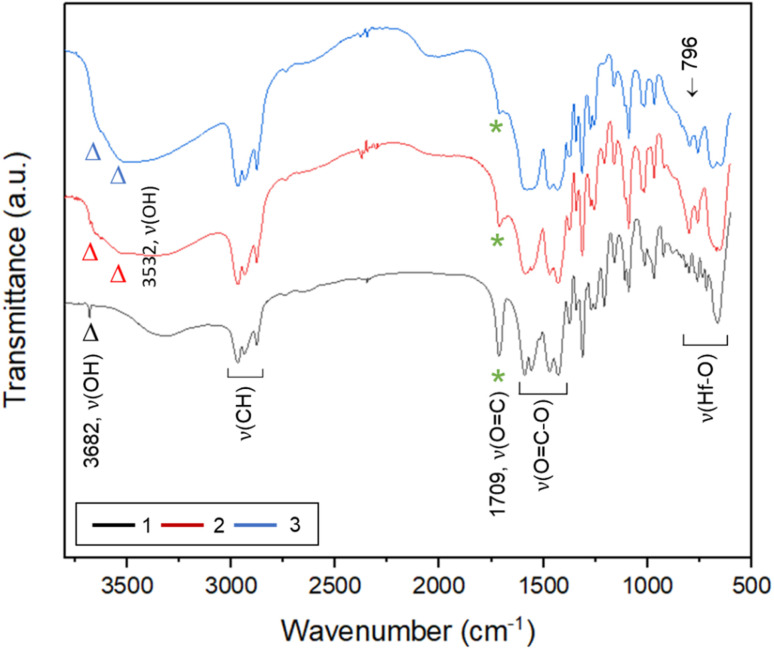
A comparison of IR spectra of clusters 1–3.

As shown in Scheme 1, cluster 1 was treated with LiOH (0.05 M) in DCM/H_2_O (2 : 1) (DCM: dichloromethane) in a stainless steel vessel at 40 °C for 12 h, forming a suspension in which an insoluble solid was removed with filtration. The solution was further extracted with DCM; the extracts were washed with H_2_O. Crystallization of the crude product in DCM/hexane afforded a crystalline solid, which unfortunately did not show diffraction patterns with X-ray diffraction studies. This new cluster is postulated to have the formula Hf_6_O_4_(OH)_8_(*s*-BuCO_2_)_8_, according to two elemental analysis data sets: calcd: C: 23.10%, H: 3.88%; found: C: 23.02%, H: 4.19%; and C: 23.11%; H: 4.17%. This formula is related to starting cluster 1 by substitution of four carboxylate ligands with four hydroxide ligands in the core structure of Hf_6_O_4_(OH)_4_(*s*-BuCO_2_)_12_. Thermogravimetric analysis (TGA) was also performed to support this chemical formula. Fig. 1 shows the TGA curve of cluster 3, which shows a steady loss of 20 wt% over the range 100–350 °C. A loss of H_2_O and *s*-BuCO_2_H is likely to occur according to our IR study (Fig. 2). A second stage is indicated by the 350–500 °C range, resulting in a 17.1% weight loss. A typical *s*-BuCO_2_Hf decomposition is likely to occur in this second stage. Our proposed formula will predict a 60.7 wt% for the formation of HfO_2_ residues, very close to the experimental data (*ca.* 62.9 wt%).

To ensure that cluster 3 has the same frameworks as clusters 1 and 2, their IR absorption bands in a KBr pellet are depicted in Fig. 2. Notably, clusters 2 and 3 have nearly the same IR absorption characters throughout the whole absorption region. Notably, the strong *ν*(C=O) band at 1709 cm^−1^ is due to three carboxylic acids embedded in cluster 1 whereas this band is weak for clusters 2 and 3, probably due to a small hydrolysis of an *s*-BuCO_2_Hf ligand in the KBr pellet. The sharp band at 3682 cm^−1^ of clusters 1–3 is assignable to the *ν*(OH) stretching mode of Hf_3_(OH).^25,26^ But a new *ν*(OH) band at 3532 cm^−1^ appears only for clusters 2 and 3, which can be assignable to Hf_2_(OH); this assignment is acceptable because its associated *ν*(Hf–O) stretching peak appears at 796 cm^−1^. The resemblance in their IR absorption characters suggests that all three clusters may have the same frameworks. We also made a comparison of the ^1^H NMR spectra of three clusters 1–3, and again the close resemblance in the NMR patterns and chemical shifts (see Fig. S7†) indicates the same structural frameworks for all clusters.

**Fig. 3:**
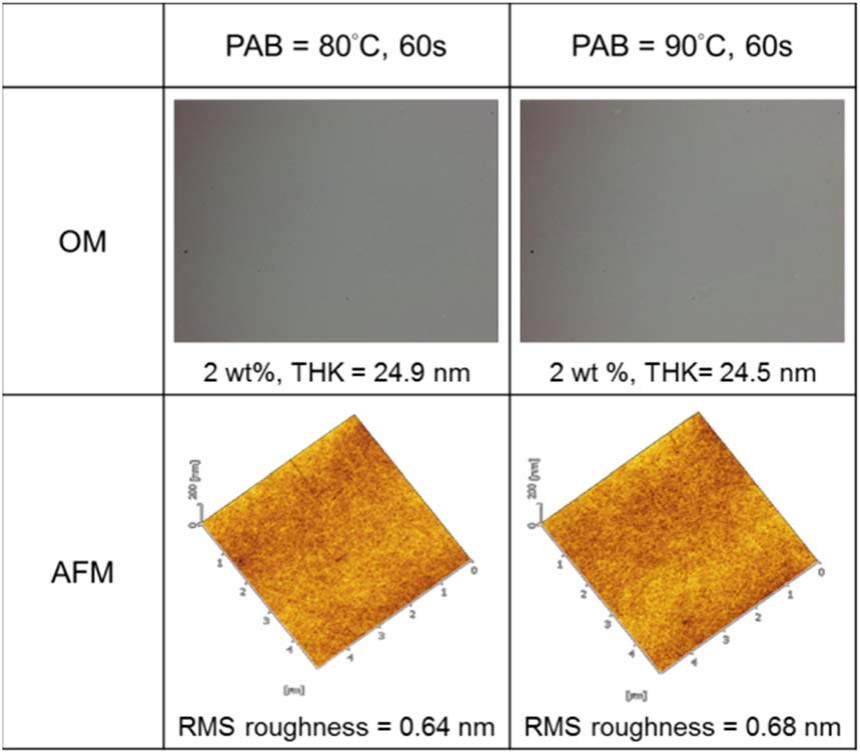
Surface films of cluster 3 after solution spin-casting.

Thin films of cluster 3 have been characterized with an optical microscope (OM) and atomic force microscope (AFM); the images are provided in Fig. 3. The films were prepared at 2.0 wt% in 4-methyl-2-pentanol before spin coating at a speed of 1500 rpm for 10 s and 2000 rpm for 25 s. PAB (post apply bake) was performed at 80 °C and 90 °C for 60 s before cooling to RT for 24 h. Cluster 3 shows no visible defects over a 500 μm × 600 μm domain, as shown by OM images. With a 24 nm thickness, cluster 3 is very smooth in the film surface with roughness *ca.* 0.64 nm and 0.68 nm at PAB at 80 °C and 90 °C respectively.

**Fig. 4:**
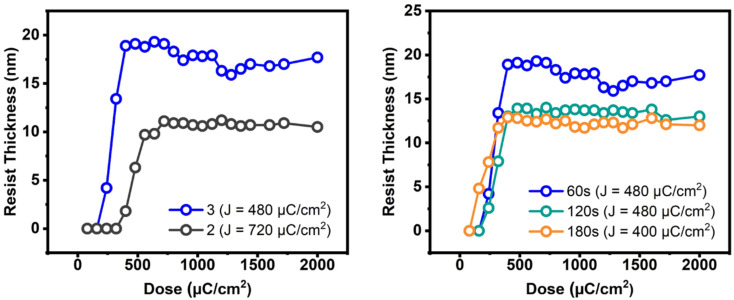
E-beam contrast curves: left figure: 2.5 wt%, initial height 30.2 and 32 nm for clusters 3 and 2 respectively. Right figure: 2.5 wt% initial thickness 30.2 nm, PAB 80 °C, 60–180 s.

E-beam studies were used as tools to estimate the photosensitivity of cluster 3; cluster 2 was used as a reference. Fig. 4 (left) shows the e-beam contrast curves, which show the remaining fraction of the exposed resist, after treatment with a developer after exposure. The curves were obtained as a function of e-beam doses. These contrast curves indicate typical patterns for negative-tone photoresists. For cluster 2, the curve reaches a maximum at 720 μC cm^−2^, but small e-beam doses of *ca.* 400 μC cm^−2^ are observed for the new cluster 3. The superior sensitivity of species 3, as reflected by the slope, is due to its increasing hydroxide content. One advantage of cluster 3 is a shrinkage in film thickness, notably at a 40% level, relative to its initial height (30.2 nm). The loss of thin film thickness for its reference cluster 2 is up to 67%. With these inspiring low energy doses, the e-beam lithographic patterns are expected to be satisfactory for cluster 3 (*vide infra*).

The effects of PAB on e-beam contrast curves are studied; the results are shown in Fig. 4 (right). A thick film (32 nm) was prepared using a 2.5% solution in 4-methyl-2-pentanol. This film was baked at 80 °C for 60 s, 120 s and 180 s before being stored under nitrogen. The respective maxima at three conditions are found at 450 μC cm^−2^, 450 μC cm^−2^ and 400 μC cm^−2^ for the intervals at 60 s, 120 s and 180 s. The photosensitivity, as reflected by the three slopes, is in the order: 60 s > 120 s = 180 s. We postulate that a prolonged PAB (*t* = 180 s) induces a Hf-OH dehydration in addition to a partial removal of carboxylic acid (*s*-BuCO_2_H) from an *s*-BuCO_2_Hf ligand; the latter is indicated by IR spectra (see Fig. 2). These actions likely save E-beam energy at a long PAB interval because some molecular aggregations can occur before e-beam exposure. We initially conducted a trial of e-beam lithography using an extended PAB treatment. However, the results showed poor contrast in the lithography. After the standard pattern development, we measured the thickness of the unexposed film, which was approximately 4 nm in thickness. It is believed that the extended PAB treatment reduced the film's solubility to the developer, and this hypothesis also accounts for the reduced thickness of films baked for 120 and 180 seconds (as illustrated in Fig. 4, left).

**Fig. 5:**
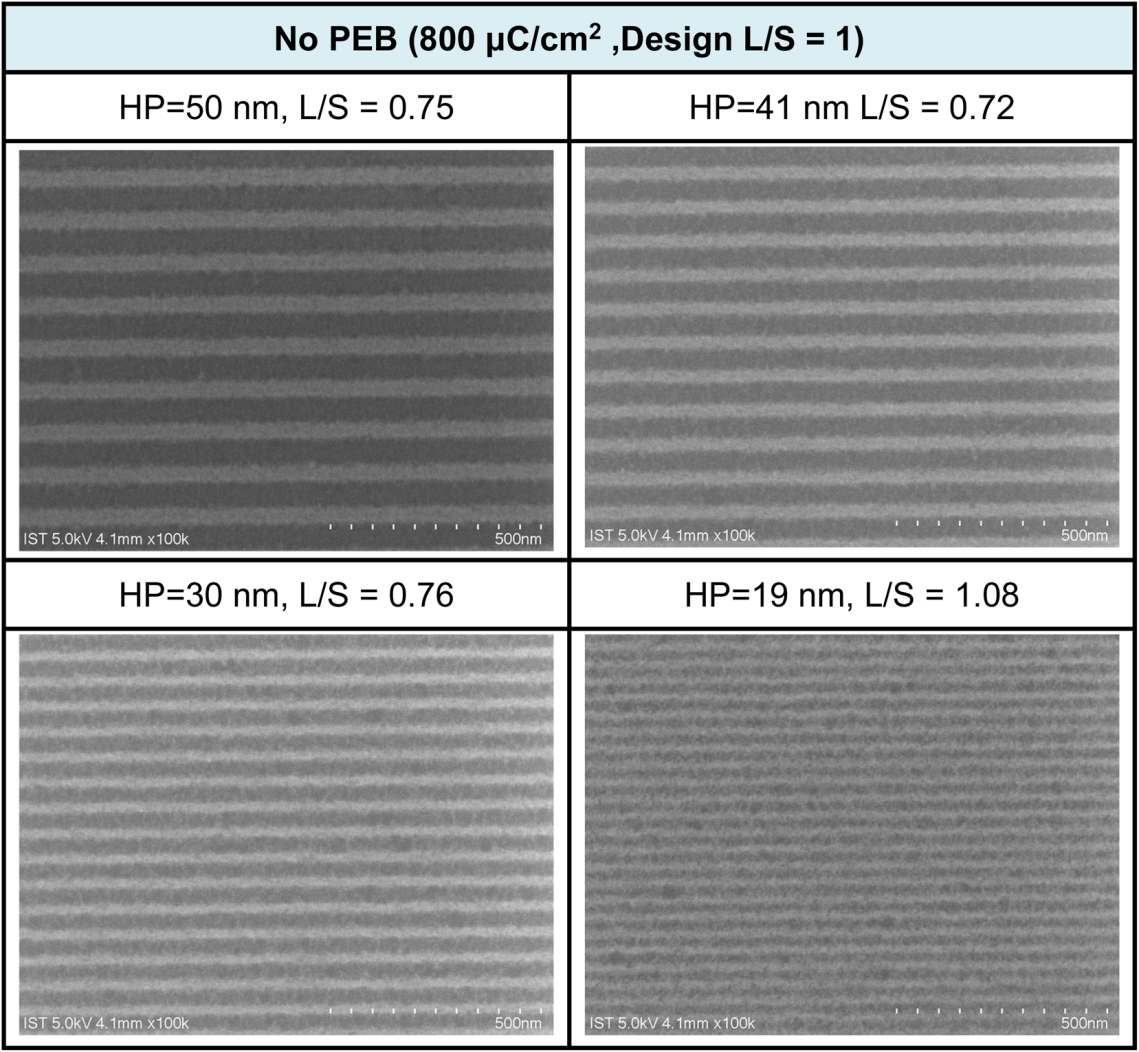
SEM images of e-beam lithographic patterns for cluster 3 at different doses and half-pitches; the design is L/S = 1 : 1, developer: hexane/2-heptanone 1 : 1, 60 s. Initial film thickness 24.5 nm.

The e-beam lithographic patterns of cluster 3 were developed with a 24.5 nm thickness using the developer (hexane/2-heptanone = 1/1, 60 s). PAB at 80 °C (60 s) was selected because the film baked for 180 s remains 3–4 nm in thickness after the pattern development. Fig. 5 shows the SEM images of the e-beam patterns of cluster 3 with small HP = 19–20 nm under small e-beam doses (800 μC cm^−2^); no PEB (post exposure baking) occurs here. A drastic improvement is noted for cluster 3, as compared to the reference cluster 2, which only reaches an HP = 30 nm pattern with a high dose of 1760 μC cm^−2^. We believe that a small loss of film thickness might be critical to achieve a high resolution pattern. Cluster 2 is only accessible to an HP = 31 nm pattern due to a 67% loss of film thickness. In Fig. 5, the L/S values (L = line; S = space) are estimated to be within 0.72–0.75 for the HP = 50–30 nm e-beam patterns, but increase to L/S = 1.08, due to photoresist blurring in small domains.

**Fig. 6:**
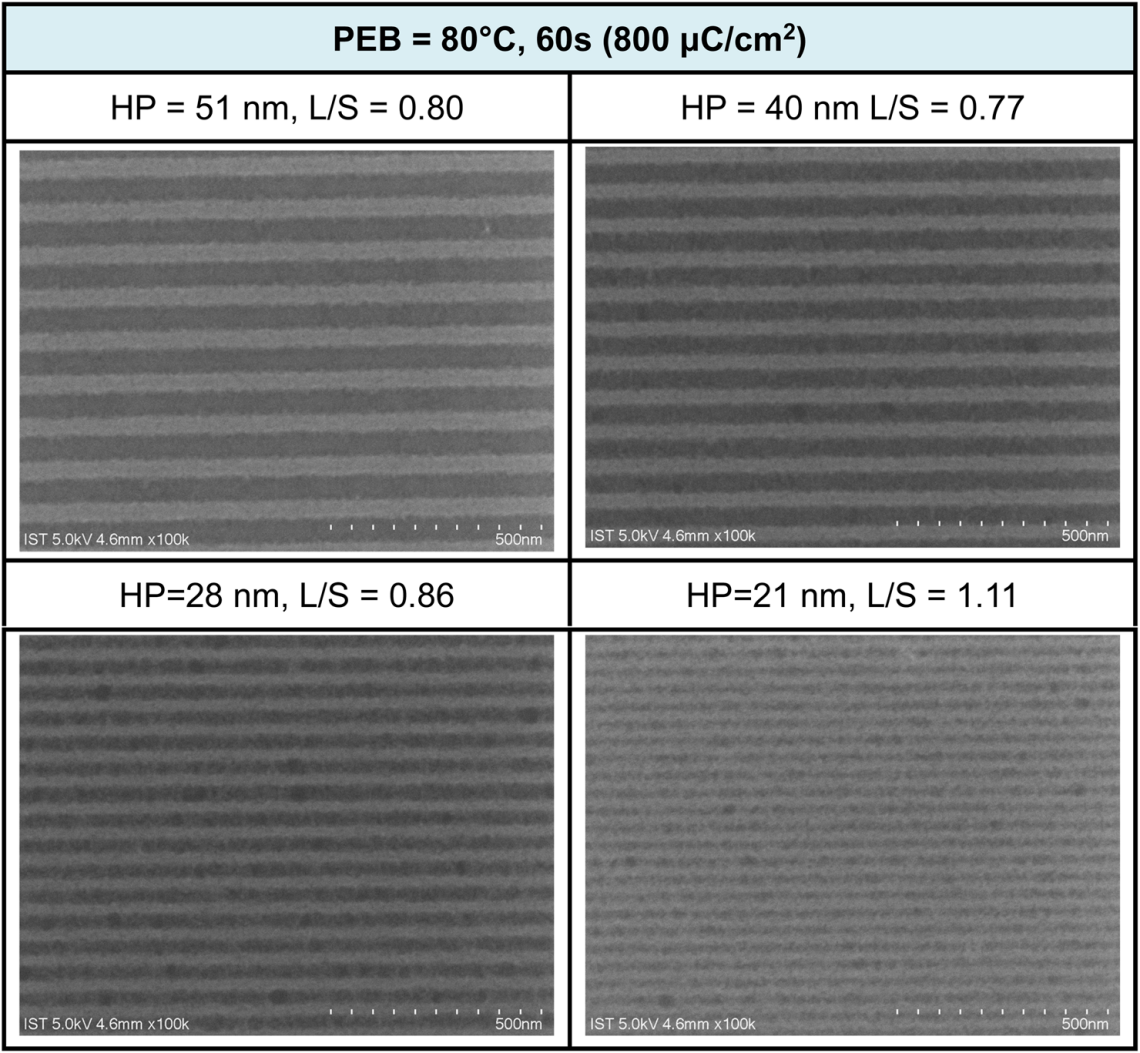
SEM images of e-beam lithographic patterns for cluster 3 at different doses and half-pitches; the design is L/S = 1 : 1. PEB (80 °C, 60 s), developer: hexane/heptanone 1 : 1, 60 s. Initial film thickness 24.5 nm.

Additional e-beam patterns are presented in Fig. 6, along with a PEB (post-exposure baking) treatment (80 °C, 60 s). It is worth noting that the additional PEB procedure does not result in energy savings, as comparable doses of approximately 800 μC cm^−2^ are still required for pattern development. Within the various e-beam patterns, with HP ranging from 28 to 50 nm, the corresponding L/S values were calculated to be in the range of 0.77 to 0.86 nm. However, the value is quickly increased to L/S = 1.1 for the smaller HP = 21 pattern. These L/S values are slightly larger than those observed without PEB, possibly due to a lower degree of Hf-OH dehydration. The patterns depicted in Fig. 5 and 6 exhibit characteristics of photolytic decarboxylation, where the smaller HP (HP = 21 nm) results in photoresist blurring with a larger L/S value of 1.1. In our forthcoming EUV study, this PEB treatment significantly impacts the EUV energy doses. Additional SEM images of e-beam patterns at different half-pitches under various e-beam doses can be found in the ESI (refer to Fig. S1 and S2).†

**Fig. 7:**
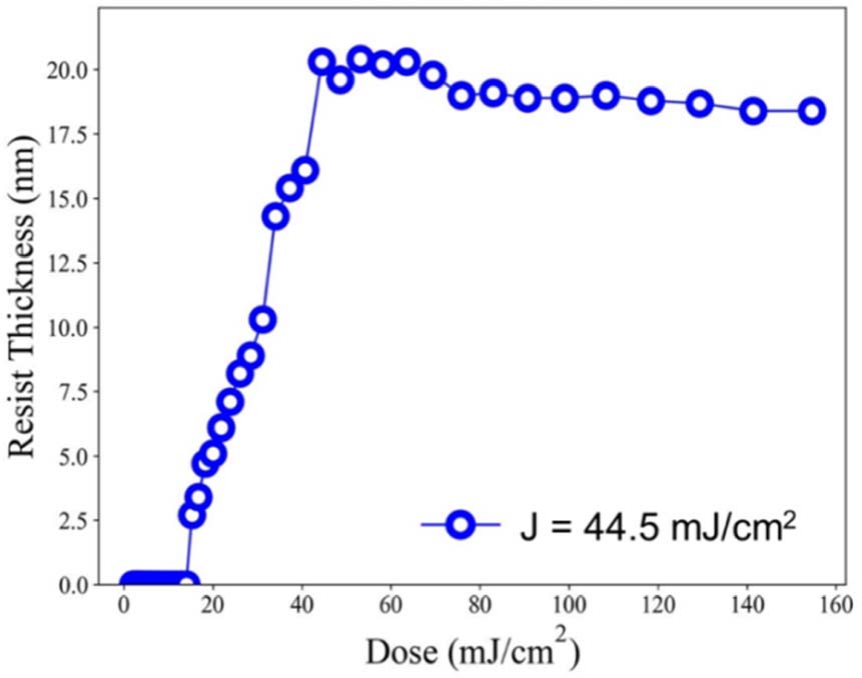
EUV contrast curve; no PEB, initial thickness 24.5 nm.

Our ultimate goal is to create high-resolution EUV patterns using small energy doses. The EUV exposure experiments were conducted at the Swiss Paul Scherrer Institute EUV Center (PSI) using EUV light at 13.5 nm. When working with a film thickness of 24.5 nm, the exposure contrast curve demonstrates an increase with the rise in EUV doses, starting at 18.5 mJ cm^−2^ and rapidly reaching its peak at 20.8 nm at a dose of 44.5 mJ cm^−2^, as illustrated in Fig. 7. We are pleased to state that this critical energy requirement is significantly lower than that of the reference sample 2, which stands at 75 mJ cm^−2^. In comparison to the initial height of 24.5 nm, we observed only a 13% reduction in film thickness. Consequently, it can be inferred that cluster 3 is more photosensitive than its reference cluster 2 in both EUV and e-beam energy doses.

**Fig. 8:**
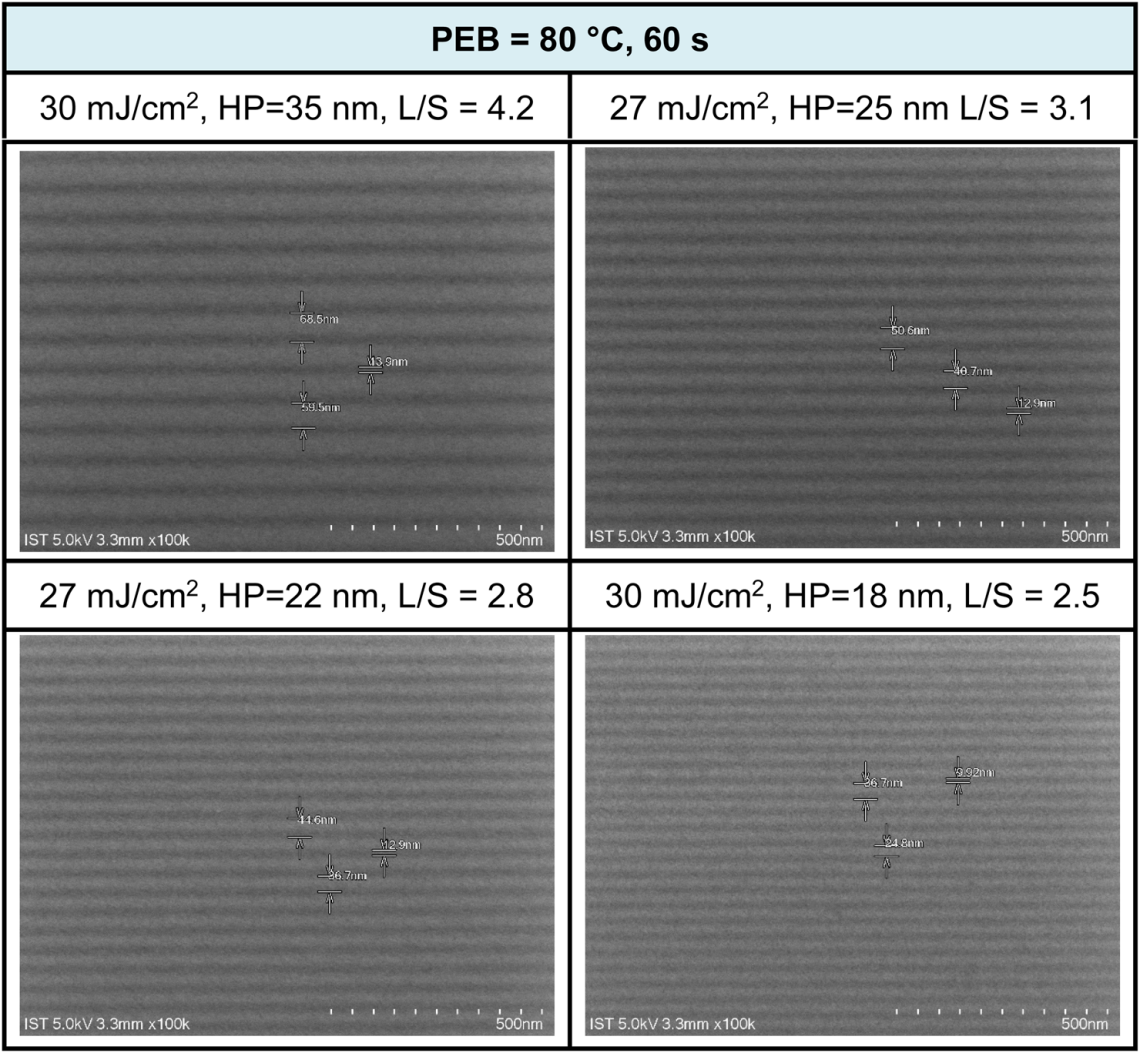
SEM images of EUV lithographic patterns: THK = 24.5 nm, PAB (80 °C, 60 s, THK = 24.5 nm); developer: hexane/heptanone 1 : 1, 60 s.

**Fig. 9:**
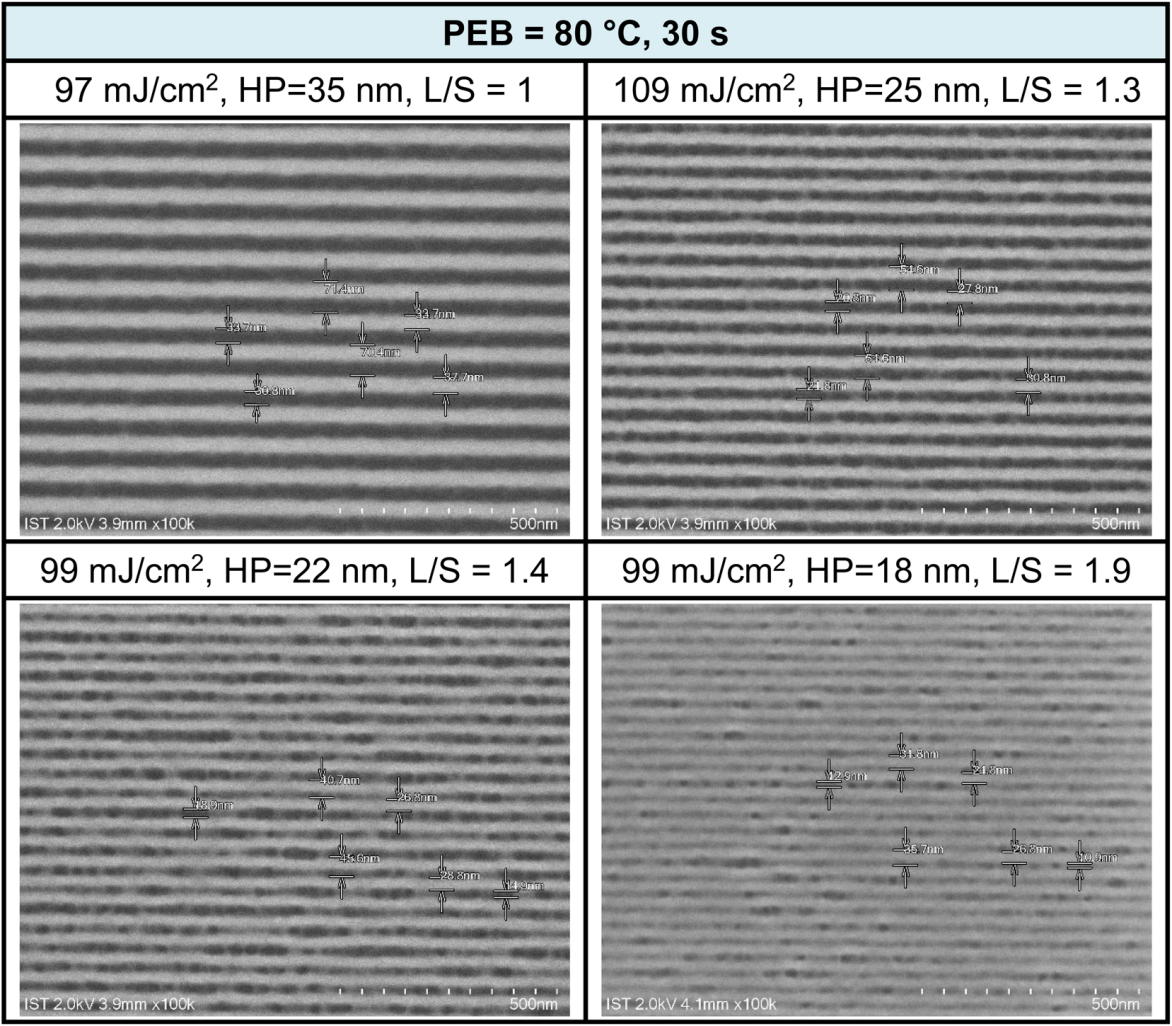
SEM images of EUV lithographic patterns: THK = 24.5 nm, PAB (80 °C, 60 s, THK = 24.5 nm).

We conducted the development of high-resolution patterns using PEB at different time intervals (30 and 60 s at 80 °C). The developer was a mixture of hexane and 2-heptanone in a 1 : 1 ratio, with a 60 second cleaning step. SEM images of EUV patterns under two different PEB durations (60 and 30 s) are presented in Fig. 8 and 9. PEB plays a crucial role in reducing EUV doses as it facilitates molecular aggregation in negative-tone photoresists due to thermal activation. A notable example is the tin cluster (BuSn)_12_O_15_(OH)_6_X_2_ (ref. 19–22) (X = Cl, OH, and carboxylate), where PEB aids in the dehydration of their six Sn-OH groups, resulting in the formation of three Sn–O–Sn units. Cluster 3 possesses eight Hf-OH groups, making us opt for an extended PEB interval (80 °C, 60 s) to achieve a reduction in EUV energy doses.

EUV exposure was carried out using an interference mask featuring dense line/space patterns with half pitches ranging from 17 to 50 nm. In the case of our new cluster 3 with a 60 second PEB interval, a pattern begins to form at a low energy level of *J* = 10 mJ cm^−2^. However, a well-defined pattern is achieved more effectively with doses in the range of *J* = 27–30 mJ cm^−2^. Fig. 7 illustrates a series of SEM images, showcasing resolutions for HP values of 35, 25, 22, and 18 nm. Additional SEM images at different EUV doses can be found in the ESI (Fig. S3).† The EUV patterns in Fig. 7 exhibit significant top loss due to the relatively high threshold energy in the EUV contrast curve, approximately 44.5 mJ cm^−2^. This top loss is because the exposed photoresist is not fully mature and remains partially soluble in the developer. An interesting observation is the line/space parameters (L/S), which measure 4.2, 3.1, 2.8, and 2.5 for the HP values of 35, 25, 22, and 18 nm patterns, respectively. This trend is typical for most EUV photoresists which tend to exhibit increasing L/S values with smaller HP patterns, primarily due to the occurrence of photoresist blurring at smaller domains.

EUV exposure was also conducted using a brief PEB interval (80 °C, 30 s), which necessitated high EUV doses of 90–110 mJ cm^−2^. The practice of employing an extended PEB interval to lower EUV doses is well established, and is primarily attributed to the dehydration of two M–OH groups. Scumming of the photoresist was observed in the case of smaller HP values, such as 22 and 18 nm, and this phenomenon was partially influenced by the solvent developer. To mitigate this scumming issue, additional EUV exposure, a change in the solvent developer, or a reduction in film thickness can be considered. Further analysis of L/S values yielded the following results: L/S = 1.0, 1.3, 1.4, and 1.9, respectively, for HP values of 35, 25, 22, and 18 nm. This L/S trend aligns with the typical behavior of EUV photoresists. The use of a short PEB interval increases energy doses due to the relatively limited degree of molecular aggregation within this brief PEB period. Additional SEM images taken under this PEB interval (80 °C, 30 s) can be found in the ESI (Fig. S4).†

**Fig. 10:**
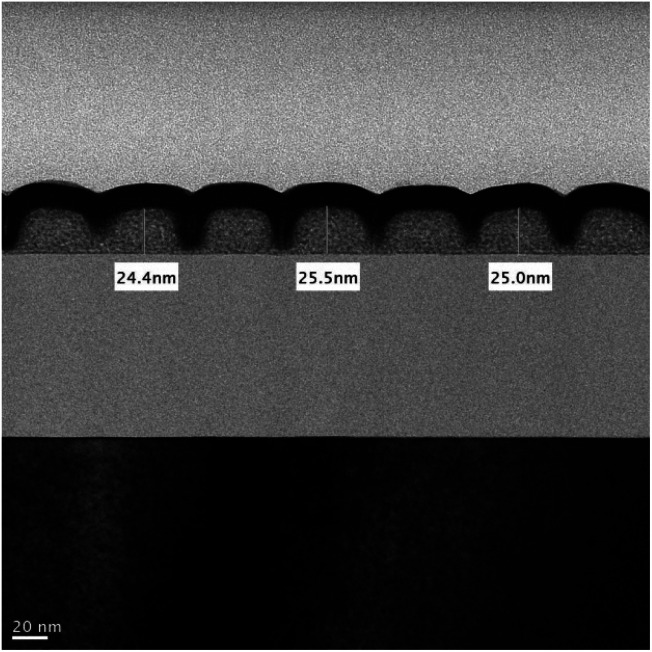
Cross-section TEM image of an HP = 25 nm pattern (*J* = 109 mJ cm^−2^).

Cross-section analysis with TEM images was performed on an HP = 25 nm pattern as shown previously in Fig. 9 (top right). The initial height of this photoresist is estimated at 24.5 nm, which is nearly the same as the thickness (24–25 nm) in this TEM image after lithographic development (see Fig. 10). Negative tone photoresists typically suffer significant shrinkage in the PEB process,^24^ but we observed nearly no loss of film thickness in this brief PEB interval (80 °C, 30 s) during the development.

**Fig. 11:**
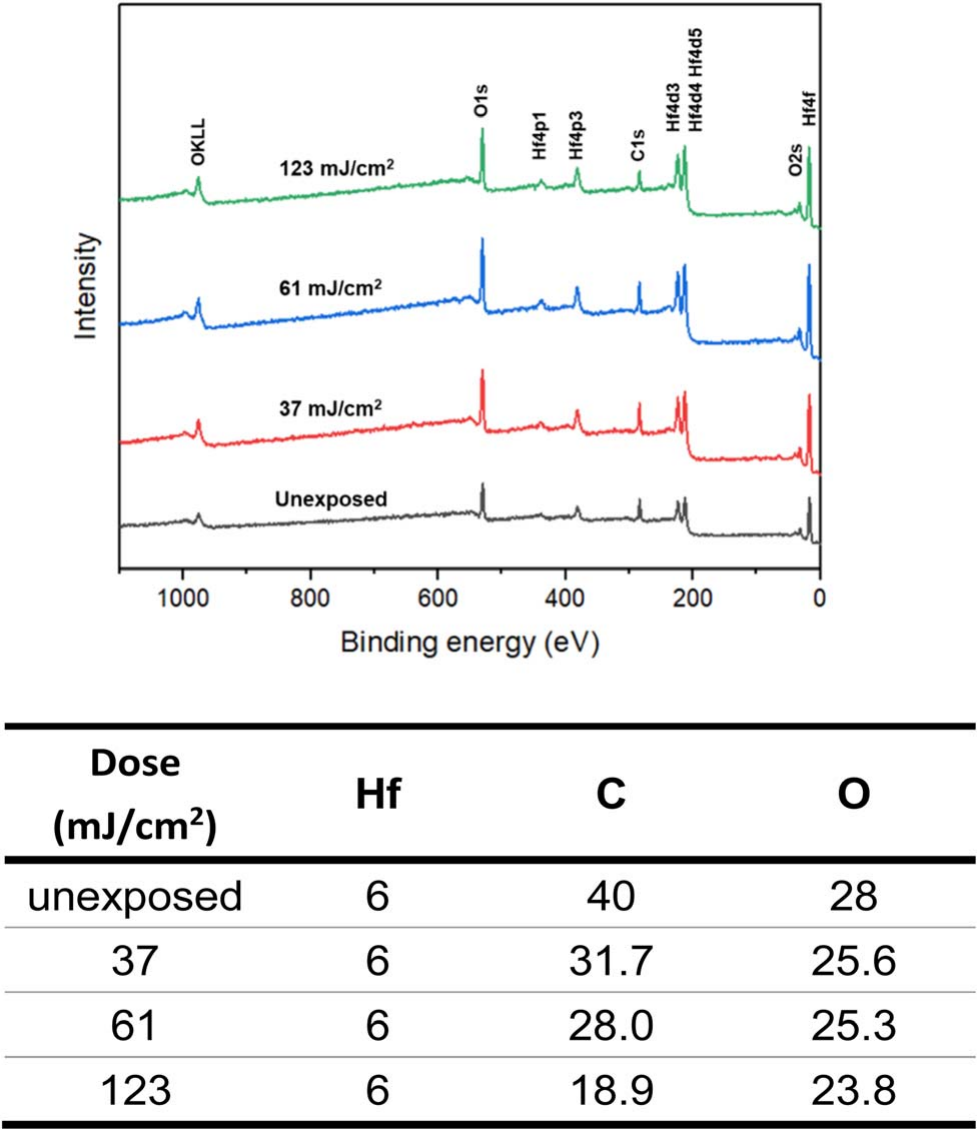
HRXPS spectra and element compositions under EUV doses.

**Scheme 3:**
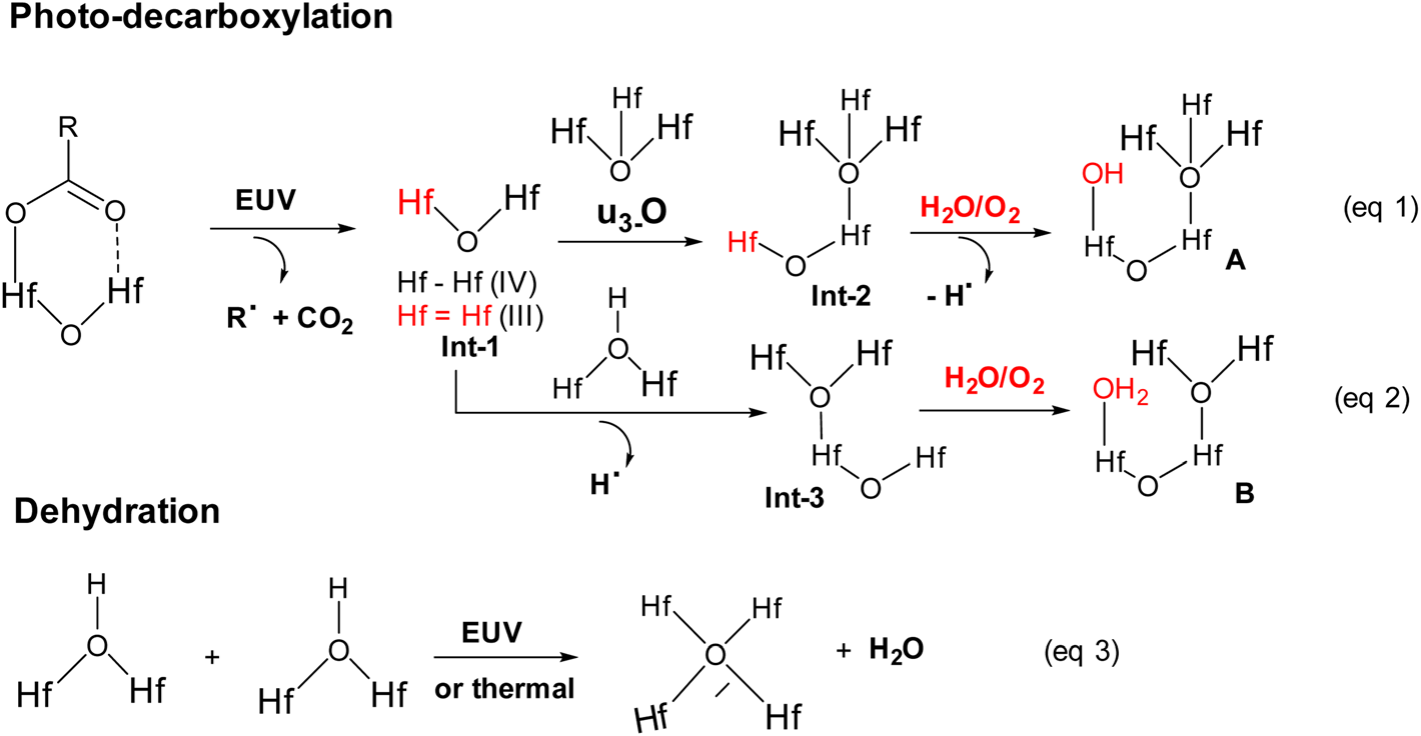
Proposed mechanism for molecular aggregation.

High resolution X-ray photoelectron spectroscopy (HRXPS) of the thin film of cluster 3 (THK = 32 nm) was performed at different EUV doses (*J* = 0, 37, 61 and 123 mJ cm^−2^). The PAB was conducted at 80 °C (60 s) before EUV exposure. These HRXPS experiments were performed without PEB so that only photo-aggregation is mainly involved. This HRXPS study is to examine the composition change of elements involving hafnium, carbon and oxygen under different EUV doses. As shown in Fig. 11, a decrease in oxygen and carbon contents is observed with increasing EUV doses; a loss of carbon content indicates a photolytic decarboxylation. Nevertheless, the loss of oxygen content is relatively small as compared to carbon content throughout all different EUV doses. In the case of *J* = 37 mJ cm^−2^, a loss of 8.3 carbon atoms corresponds to a decomposition of 1.7 molecules of *s*-BuCO_2_Hf. The oxygen loss should be around 3.4 atoms for a typical RCO_2_ → R˙ + CO_2_, which is actually larger than our observed 2.4 oxygen atoms. This outcome is indicative of a prior photolytic decarboxylation, followed by H_2_O/O_2_ exposure before forming species A or B (eqn (1) and (2), Scheme 3). Species A or B are expected to lose only one oxygen for the photolytic decomposition of one *s*-BuCO_2_Hf group. Upon EUV-induced decarboxylation, the resulting intermediates Int-1 have two empty sites to further react with μ_3_-oxide atoms to yield species Int-2, ultimately yielding species A after exposure to air. Alternatively, intermediate Int-1 can undergo a redox reaction with Hf_2_OH, forming an aggregation intermediate Int-3, finally affording species B after air exposure. Loss of extra oxygen atoms at EUV light *J* = 37 is probably attributed to a Hf-OH dehydration as depicted in eqn (3). A combined action of a Hf-OH dehydration with two decarboxylations in eqn (1) and (2) well rationalizes our HRXPS studies.

HRXPS is also used to estimate the mass loss at *J* = 37 mJ cm^2^; a loss of 8.2 carbon atoms and 2.4 oxygen atoms is observed in Fig. 11. Such a mass loss corresponds to a total loss of 138 g mol^−1^ for cluster 3 that has a molecular weight of 1381.6 g mol^−1^. A 10% weight loss is obtained at *J* = 37 mJ cm^−2^. In the EUV contrast curve, we observed a loss of height of *ca.* 13% for the exposed thin film at the threshold energy *J* = 44.5 mJ cm^−2^.

**Fig. 12:**
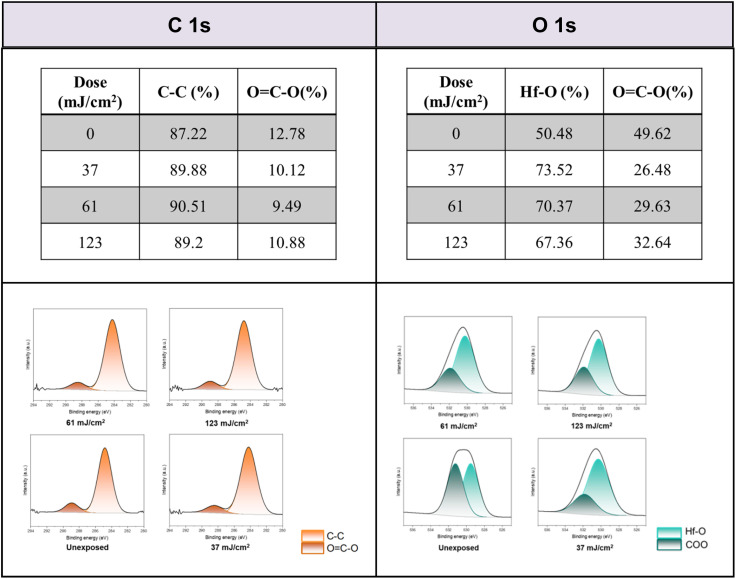
Spectra simulations on C(1s) and O(1s) XPS absorptions.

Detailed XPS simulations were conducted on the C(1s) and O(1s) absorption peaks to characterize the mechanism. Simulation spectra are shown in Fig. 12 together with their quantitative analysis. Two components were found for the observed C(1s) peaks, including the sp^3^ C–C carbons and CO_2_ components centered at 284 and 289 eV respectively. Their relative ratios show little change at different EUV doses *J* = 37 → 123 mJ cm^−2^, showing a clean decarboxylation process, as in eqn (1) and (2). In the O(1s) peak analysis, the component centered at 530 eV is assignable to inorganic Hf–O species including Hf_2_OH, Hf_2_O and Hf_3_O; the second component centered at 532 eV is assignable to the *s*-BuCO_2_Hf oxygen. At *J* = 37 mJ cm^−2^, the inorganic (Hf–O) band gains intensity to 73% while the *s*-BuCO_2_Hf oxygen intensity is decreased to 27%. These data well fit our model in eqn (1) and (2) that *s*-BuCO_2_Hf was photo-decomposed to inorganic Hf–O species A or B. Notably, with increased *J* = 37 → 123 mJ cm^−2^, the inorganic Hf–O component starts to lose intensity while the Hf_2_(O_2_C–^*s*^Bu) band gains intensity. We postulate that a Hf-OH dehydration, as depicted in eqn (3), is still occurring at high EUV doses. Our photolytic decarboxylation model together with a Hf-OH dehydration, as depicted in eqn (1)–(3), well fit these HRXPS simulation spectra.

**Fig. 13:**
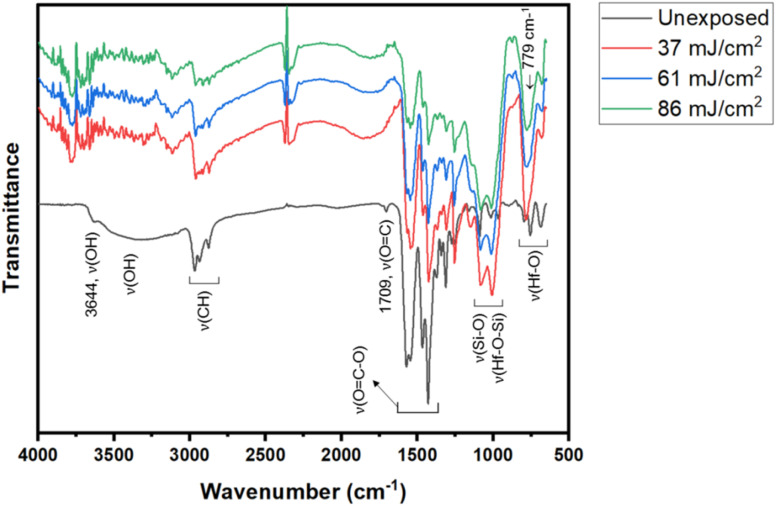
FTIR spectra at different EUV doses; the intensities at *J* = 37–86 mJ cm^−2^ were enlarged by 6 fold to show clarity.

FTIR in ATR (attenuated total reflectance) mode was used as a tool to study surface composition under EUV light. A film was prepared with 2.5 μm thickness before a PAB process (80 °C, 60 s). After EUV exposures at 0, 37, 61 and 86 mJ cm^−2^, we were unable to locate the EUV exposure area with our vision. Accordingly, the films were developed with the same developer (2-heptanone/hexane) for 10 s to show the EUV exposure area. The films were dried at 80 °C for 60 s to remove solvent residues. In the unexposed film, there is a small peak of free RCO_2_H with *ν*(C=O) 1709 cm^−1^, but this band is not observed with EUV exposure. Before the PAB baking, water residue is embedded with cluster 3, and hydrolysis of Hf_2_(*s*-BuCO_2_) might occur at the baking temperature (80 °C). The IR absorption bands at *J* = 37–86 mJ cm^−2^ were scaled up by 6 fold to show the clarity; some bands at 1000–1100 cm^−1^ are partly due to SiO_2_ absorption as the film surface is partly cracked. In Fig. 13, the spectra of unexposed film 3 have large *ν*(OH) absorptions in the 3333–3600 cm^−1^ range. With a 37 mJ cm^−2^ dose, this *ν*(OH) band greatly decreases in intensity whereas *ν*(C–H) and *ν*(CO_2_) bands at 2800–3000 cm^−1^ and 1600–1450 cm^−1^ are still strong. A Hf-OH dehydration is evident here. With increasing EUV doses, the *ν*(C–H) and *ν*(*s*-BuCO_2_Hf) bands decrease in intensity at the same pace. Notably, the inorganic *ν*(Hf–O) band at 798 cm^−1^ is relatively strong in IR absorption as compared to *ν*(C–H) and *ν*(*s*-BuCO_2_Hf) bands. Accordingly, the model in eqn (1) and (2) well rationalizes these data that a photolytic decomposition of one *s*-BuCO_2_Hf unit forms one inorganic Hf–O unit. The involvement of photolytic decarboxylation (eqn (1) and (2)) as well as a Hf-OH dehydration (eqn (3)) is again manifested by these FTIR spectra.

**Fig. 14:**
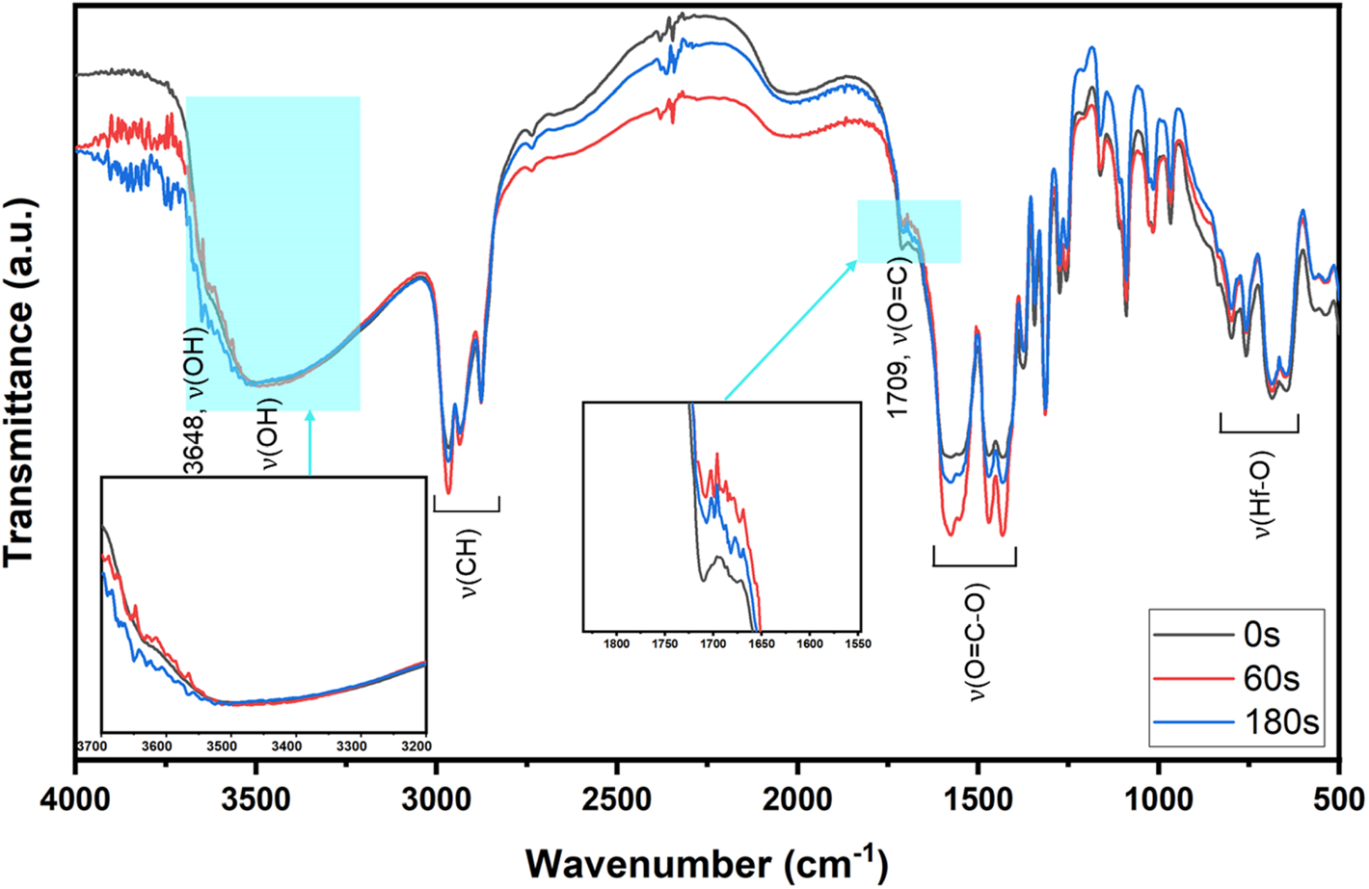
FTIR spectra of cluster 3 in KBr at different time at 80 °C.

The Hf-OH dehydration in cluster 3 was also examined by FTIR spectra over various PAB intervals. Samples were baked at 80 °C for 60, 120 and 180 s before being moved into nitrogen-filled glassware for cooling. Their KBr pellets were prepared under air as dehydration is shown to be irreversible (*vide infra*); FTIR spectra were recorded in air without special precaution. Fig. 14 shows three spectra at different intervals. None of the absorption bands including *ν*(C–H) and *ν*(O=C–O) show any noticeable change in the 3100–500 nm region. An inorganic *ν*(Hf–O) band at 796 nm^−1^ remains unchanged in IR intensity at *t* = 60, 120 and 180 s. But a loss of *ν*(O–H) intensities at *ca.* 16% and 24% is evident for *t* = 60 s and 180 s respectively. Also in Fig. 14 is a very small peak of free *s*-BuCO_2_H for all spectra; its formation arises from a slight hydrolysis of the *s*-BuCO_2_Hf ligand to form its acid form at the PAB stage. Notably, the *ν*(O–H) band at 180 s shows little change after exposure to air for 24 h. The film of cluster 3 baked at 80 °C (60 s) can be cleaned completely with 2-heptanone/hexane (60 s, 1 : 1) but the film baked for 180 s remains 3–4 nm in thickness. This fact is indicative of the irreversible nature of this Hf-OH dehydration.

Mechanistic studies with FTIR and HRXPS studies support two main aggregations as depicted in Scheme 3. First, the Hf-OH dehydration in cluster 3 can be carried out either by thermal or by EUV irradiation. A second process is a photolytic decarboxylation that only occurs with EUV light. In the HRXPS study, we observed a relatively small loss of oxygen that can be rationalized by a combined action of photolytic decarboxylation with a Hf-OH dehydration. Simulation of the C(1s) spectra indicates a clean decomposition of the *s*-BuCO_2_Hf ligand. A further analysis of the O(1s) region at *J* = 37 mJ cm^−2^ shows the formation of a new inorganic Hf–O unit at the cost of a Hf–O_2_C(*s*-Bu) unit. Further EUV irradiation (*J* = 37–123 mJ cm^−2^) is compatible with a Hf-OH dehydration. All these results well rationalize our reaction mechanism in Scheme 2. In this work, carbon radicals are generated, but such radicals are not involved in molecular aggregations as for radical acceptor ligands. Photolytic decarboxylation reactions just create two coordination vacant sites around two hafnium centers Int-1 to facilitate aggregation processes.

## Conclusions

Prior to this work, the design of negative-tone EUV photoresists relied nearly exclusively on radical chain growth in molecular aggregations. Despite the success of this design to save EUV energy, very few examples can reach high resolution patterns. This work demonstrates a new photoresist design to elude a radical chain aggregation; high resolution patterns with HP = 18 nm under 30 mJ cm^−2^ prove the viability. Synthesis of this new photoresist involves multiple hydroxide substitutions of carboxylate ligands in the well-known Hf_6_O_4_(OH)_4_(RCO_2_)_12_ clusters. Our mechanistic study reveals that EUV light not only enhances the photolytic decarboxylation, but also accelerates the dehydration of eight Hf-OH bonds. This work will inspire a large body of metal carboxylate clusters to become potential EUV photoresists after multiple hydroxide substitutions.

## Experimental section

### Material preparation and characterization

All chemicals were purchased from Sigma company. ^1^H and ^13^C NMR spectra were recorded on Bruker 400 or Bruker 500 MHz spectrometers using chloroform-*d*_1_ (CDCl_3_) as the internal standard. TGA was carried out with a Mettler-Toledo 2-HT at a heating rate of 10 °C min^−1^. FTIR spectroscopy of cluster 3 in KBr was conducted on a Bruker Vertex 80v spectrometer.

### Thin-film deposition

Cluster 3 was dissolved in 4-methyl-2-pentanol at 2.0 or 2.5 wt%; the solution was filtered through a 0.22 μm filter. The resist film was spin-coated on a SiO_2_-coated (THK 100 nm) Si-wafer at 1500 rpm for 10 s and 2000 rpm for 25 s. The wafer was baked at 80 °C for 60 s, respectively for clusters 1 and 2. The thickness of the thin films was in the range of 20.9–22.9 nm, which was measured with a J. A. Woollam model M2000. Atomic force microscopy images (AFM) were obtained with SEIKO SPA-300 HV, using the contact mode. These films were also used for e-beam and EUV exposure.

### Electron-beam lithography (EBL)

Electron-beam lithography was conducted on an Elionix ELS-7800 with an accelerating voltage of 80 kV. The beam current of 200 pA was used for the contrast curve, and 50 pA for the line pattern. After exposure, the samples were developed with 2-heptanone/hexane (1 : 1) for 60 s and rinsed with deionized water. The contrast curve of the photoresist was obtained from a series of squares (50 × 50 mm^2^), and each with different dosages ranged from 400 μC cm^−2^ to 2400 μC cm^−2^. The contrast curve was obtained by measuring the remaining thickness of each exposed square area through an α-step tool after solvent development. To analyze the resolution limit of cluster 3, different dense line features are designed from the HP ranges from 50 nm to 20 nm. The best resolution pattern was optimized at 800 and 1120 μC cm^−2^, respectively for cluster 3.

### FT-IR measurement at different EUV doses

A thin film of compound 3 was coated on a 2.5 × 5 cm^2^ silicon wafer by spreading a 4-methyl 2-pentanol solution (3.0 wt%, 0.90 mL) over this wafer, which was dried in air at room temperature for 24 h. The coated film was baked at 80 °C for 60 s. After EUV exposure, this film was developed with 2-heptanone/hexane for 10 s to show the exposed area. The wafer was then baked at 80 °C for 60 s before FT-IR measurement. The operation was conducted in air on a Bruker model Tensor 27 equipped with a KBr beam splitter. The signals were collected in transmission mode with an MCT (mercury cadmium telluride) detector; the resolution limit is 4 cm^−1^.

### Pattern development

The films after EUV exposure were baked at 80 °C for 0, or 30 or 60 s before being cooled at room temperature. The pattern was developed with 2-heptanone/hexane for 60 s before baking at 90 °C for 90 s.

### High resolution X-ray photoelectron spectroscopy (HRXPS)

HRXPS data were obtained on a ULVAC-PHI Quantera II, with a monochromatic Al Kα source (energy of 1486.7 eV). A survey spectrum was obtained with a pass energy of 280 eV with a 1 eV energy step. A pass energy of 55 eV with a 0.1 eV energy step was used to obtain the high resolution spectra of O, C, and Hf. Thin films were prepared in 32 nm thickness and baked at 80 °C for 60 s for EUV exposure at the NSRRC, Taiwan. After light exposure, standard development was performed with 2-heptanone/hexane for 60 s before drying under flowing nitrogen.

### Synthesis of cluster Hf_6_O_4_(OH)_8_(*s*-BuCO_2_)_8_

Cluster 1 (100 mg, 0.037 mmol) was transferred into a Teflon cup that was then placed with a Teflon cap before being moved into a stainless steel vessel. The sample was dissolved in dichloromethane (10 mL) before addition with a LiOH aqueous solution (0.05 M, 5 mL). The reactor was tightly locked and placed in an oven at 40 °C for 12 hours. After the reaction was complete, the solution was extracted with dichloromethane and the extracts were filtered and evaporated to dryness, affording the crude product 3 in 59% yield (45 mg, 0.022 mmol). Cluster 3 was recrystallized in a mixed solvent of dichloromethane and *n*-hexane, affording colorless crystals that have no diffraction in X-ray diffraction studies.

## Author contributions

J.-H. Liu was responsible for the design of all synthetic work. T.-S. Gau and B.-J. Lin took care of the lithographic development. P.-W. Chiu and B.-H. Chen conducted e-beam lithographic work. Y.-F. Tseng conducted the synthesis and characterization of hafnium photoresists. The chief investigator, Jui-Hsiung Liu is the passport name of Rai-Shung Liu; the patent laws of Taiwan and USA only allow passport names for patent applications.

## Conflicts of interest

The authors declare no conflict of interest.

## Supplementary Material

NA-006-D3NA00508A-s001
