# Supplementary material for: Highly hydroxylated hafnium clusters are accessible to high resolution EUV photoresists under small energy doses
Source: Nanoscale Adv. 2023 Nov 10;6(1):197–208. doi: 10.1039/d3na00508a (PMC10729921; doi:10.1039/d3na00508a)
Supplement: NA-006-D3NA00508A-s001 [file NA-006-D3NA00508A-s001.pdf]

Electronic Supplementary Material (ESI) for Nanoscale Advances.
This journal is © The Royal Society of Chemistry 2023

Supporting Information

Highly Hydroxylated Hafnium Clusters Are Accessible to High Resolution EUV Photoresists under Small Energy Doses

Yu-Fang Tseng,[a] Pin-Chia Liao, Po-Hsiung Chen*[b,c] Tsi-Sheng Gau,*[b,c] Burn-Jeng Lin,[b,c] Po-Wen Chiu [b,c] and Jui-Hsiung Liu*[a]

KEYWORDS: Hexameric Hf oxide clusters, partially decarboxylation, photoresist, EUV lithography, line/space/edge character.

[a]Department of Chemistry,
[b]TSMC-NTHU Joint Research Center
[c]Department of Electric Engineering, National Tsing-Hua University, Hsinchu, Taiwan, ROC, email: rsliu@mx.nthu.edu.tw

Content:

1. Representative Synthetic procedures -- 1
2. SEM images E-beam lithography patterns ---------------------------------- 2
3. SEM images EUV lithography patterns --------------------------------------- 4
4. Spectral data of key compounds --6
5. ^{1}H and ^{13}C NMR of key compounds -- 7

1. Representative synthetic procedures

Unless otherwise noted, all reactions were carried out under nitrogen atmosphere inoven-dried glassware using standard syringe, cannula and septa apparatus. Dichloromethane and toluene were dried over CaH_2 and distilled. Reagents were purchased from commercial sources and used without purification, unless otherwise stated. ^{1}H NMR and ^{13}C NMR spectra were recorded on a Bruker 400 MHz and Bruker 500 MHz spectrometers using chloroform-d ($CDCl_3$) as the internal standard. The ESI-Mass were perdormed using JEOL JMS-700. The EA analysis was performed by elementar vario EL cube. The TGA were performed using Mettler-Toledo 2-HT. FTIR Spectroscopy of power samples was in a Bruker Vertex 80v spectrometer. The AFM measurements were using SEIKO SPA-300HV. Electron-beam lithography was done by utilizing Elionix ELS-7800 with an accelerating voltage of 80 kV and a beam current of 200 pA. The EUV-IL system at the Swiss Light Sources (SLS), Paul Scherrer Institute, utilizes 13.5 nm EUV light. HRXPS measurements were performed in a ULVAC-PHI Quantera II, with a monochromatic Al Ka source (energy of 1486.7 eV).

3. SEM image of E-beam lithography patterns

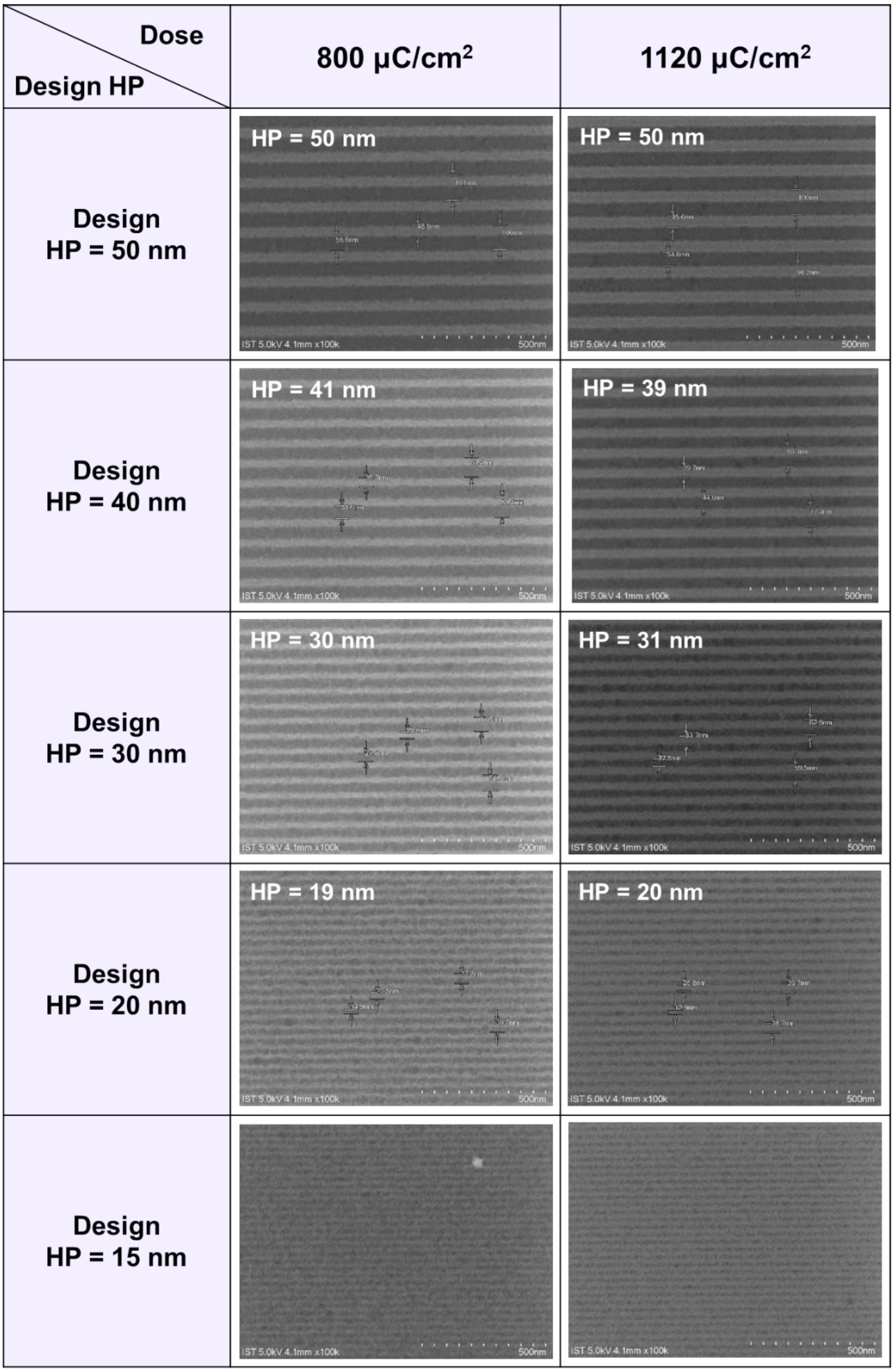

Figure S1. SEM images of E-beam lithography patterns on **3**. Process parameter: 2 wt%, THK= 24 nm, Developer: 2-Heptanone : Hexane = 1 : 1 60 s, **no PEB**

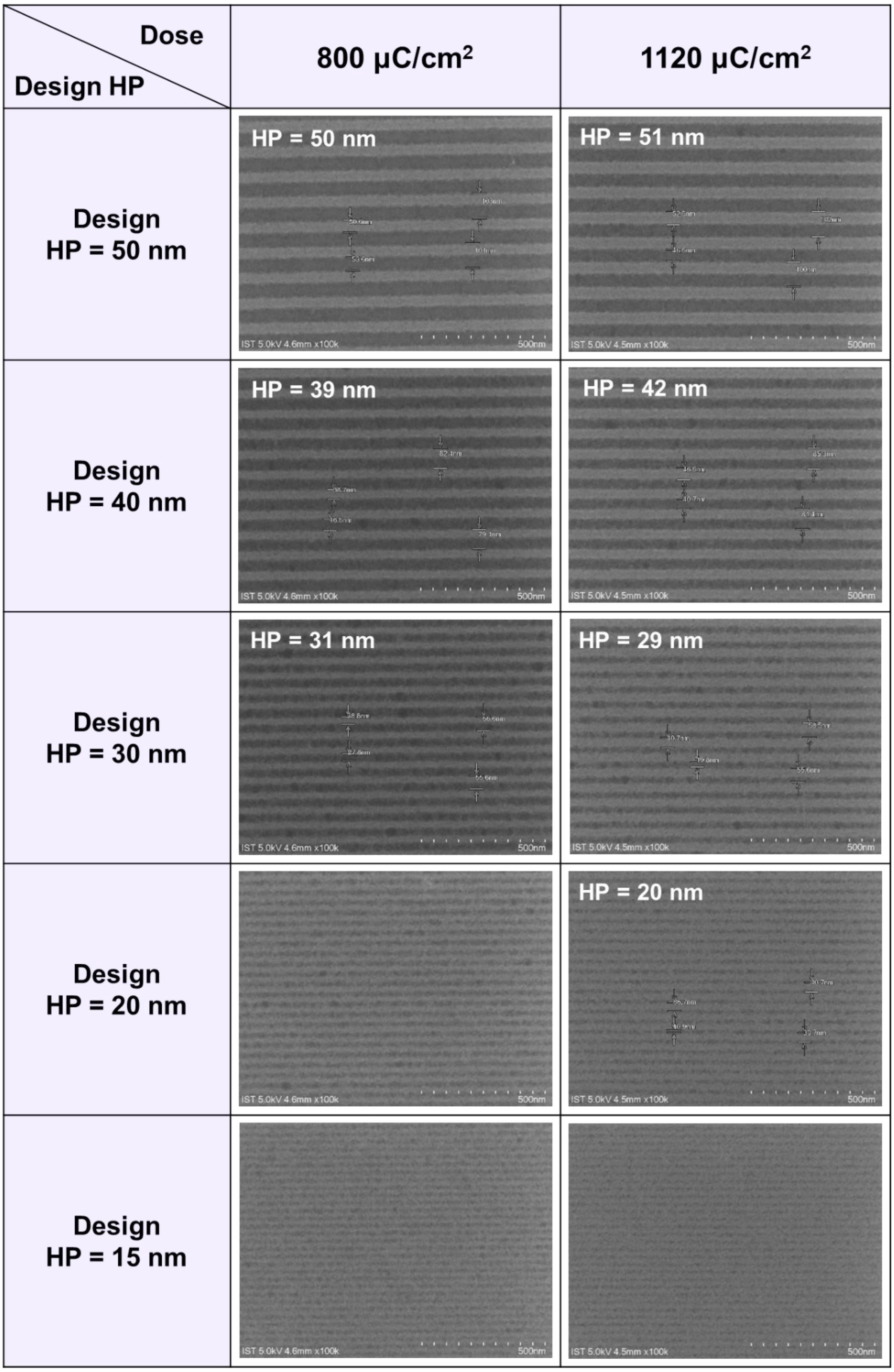

Figure S2. SEM images of E-beam lithography patterns on **3**. Process parameter: 2 wt%, THK= 24 nm, Developer: 2-Heptanone : Hexane = 1 : 1 60 s, PEB= 80°C, 60s

.

4. SEM image of EUV lithography patterns

Dose / Design HP	~ 10 mJ/cm^2	~ 30 mJ/cm^2
**Design HP = 50 nm**	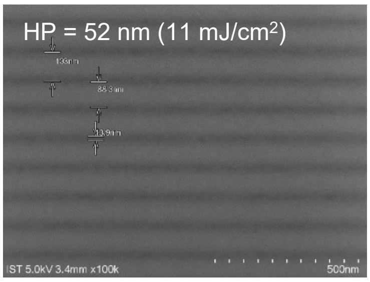	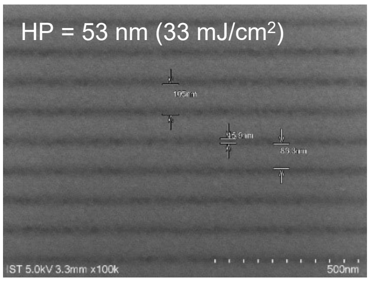
**Design HP = 35 nm**	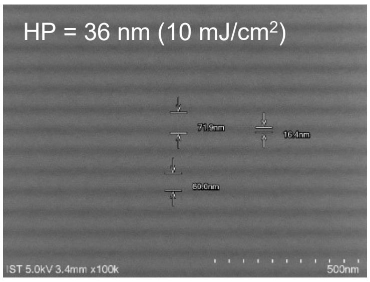	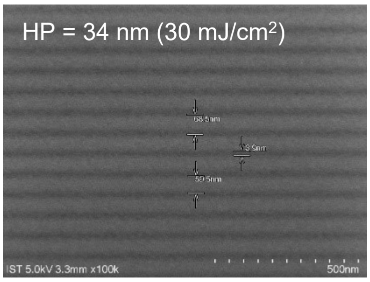
**Design HP = 25 nm**	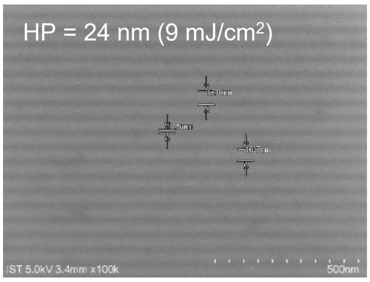	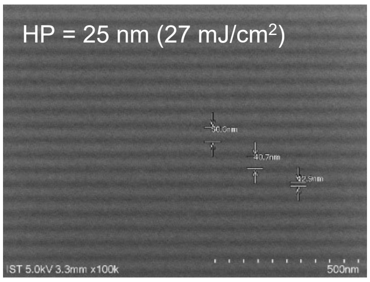
**Design HP = 22 nm**	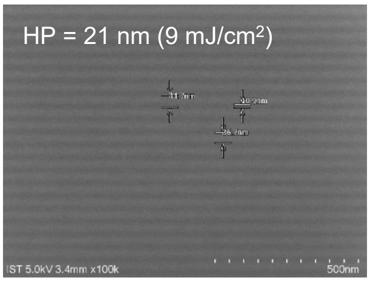	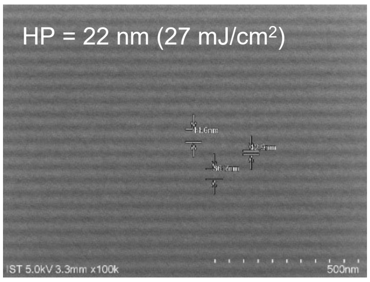
**Design HP = 18 nm**	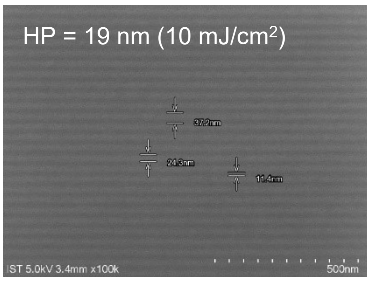	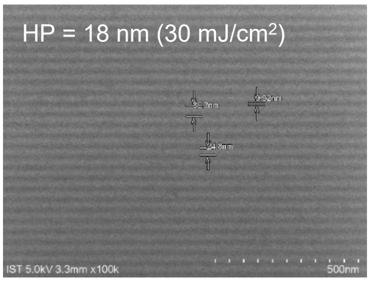

Figure S3. SEM images of EUV lithography patterns on **3** HP= 50, 35, 25, 22, 18 nm at different dose. Process parameter: 2.0 wt%, THK= 24 nm, Developer: 2-Heptanone : Hexane = 1 : 1 60 s, PEB= 80℃ 60 s

Dose / Design HP	~ 90 mJ/cm^2	~ 100 mJ/cm^2
Design HP = 50 nm	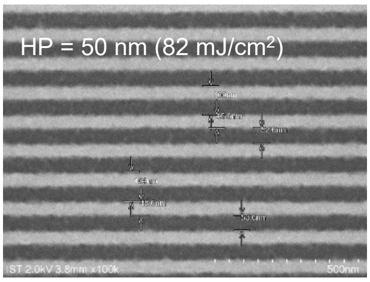	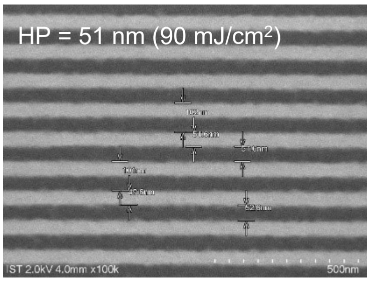
Design HP = 35 nm	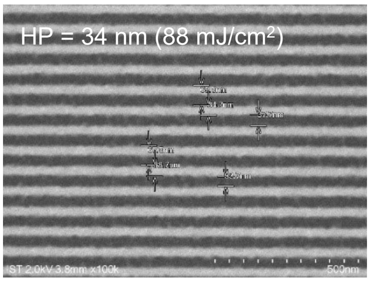	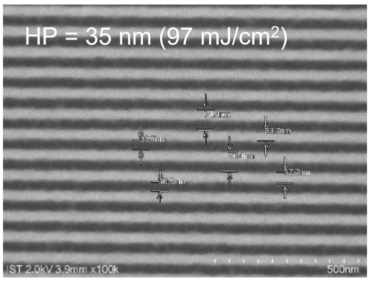
Design HP = 25 nm	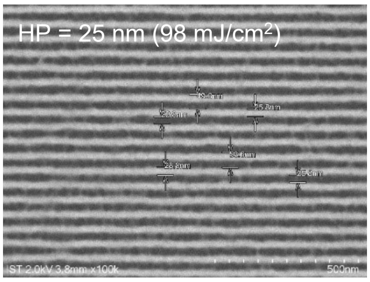	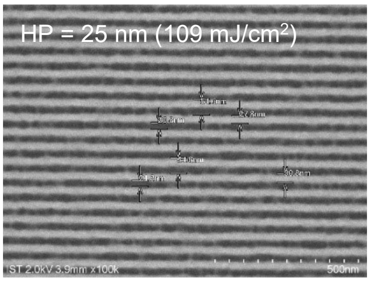
Design HP = 22 nm	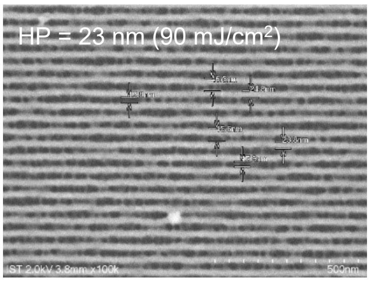	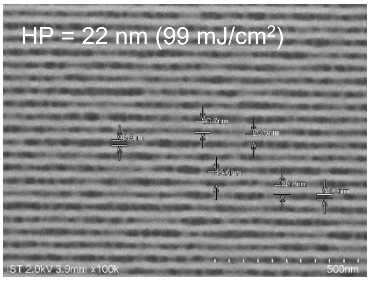
Design HP = 18 nm	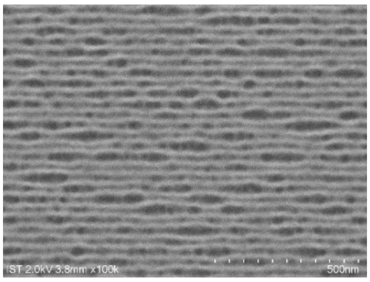	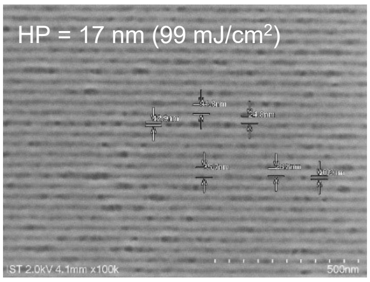

Figure S4. SEM images of EUV lithography patterns on **3** HP= 50, 35, 25, 22, 18 nm at different dose. Process parameter: 2.0 wt%, THK= 24 nm, Developer: 2-Heptanone : Hexane = 1 : 1 60 s, PEB= 80℃ 30 s

6. Spectral data of key compounds.

6.1 Spectral data for highly decarboxylated 6-Hafnium Cluster with 2-methylbutanoic acid ligand 3.

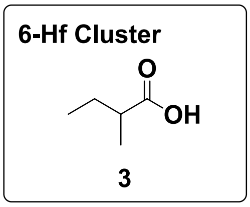

White solid (59%) ; ^{1}**H NMR** (400 MHz, $CDCl_3$) : δ 2.11 (s, 8H), 1.65 (s, 8H), 1.30 (s, 8H), 1.03 (s, 24H), 0.84 (s, 24H) ; ^{13}**C NMR** (125 MHz, $CDCl_3$) : δ 181.9, 42.8, 26.6, 16.2, 11.9. ; **EA Anal.** Calcd. for $\mathbf{C_{40}H_{80}O_{28}Hf_6}$: C : 23.10% ; H : 3.88%, found : C : 23.11% ; H : 4.17

7. ^{1}H and ^{13}C NMR of cluster 3

6-Hf Cluster

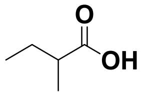

3

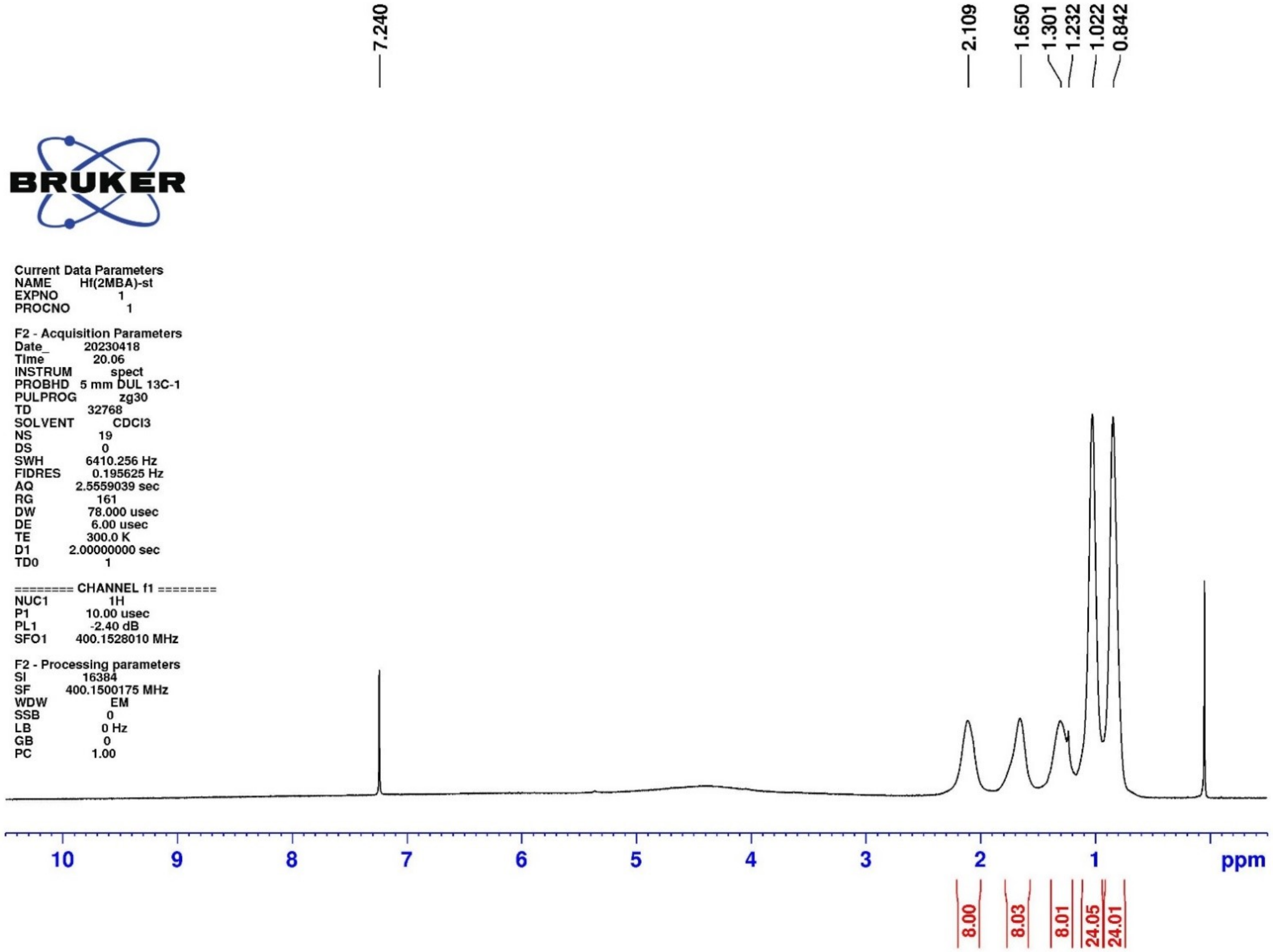

Figure S5. 1H NMR of cluster **3**

6-Hf Cluster

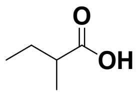

3

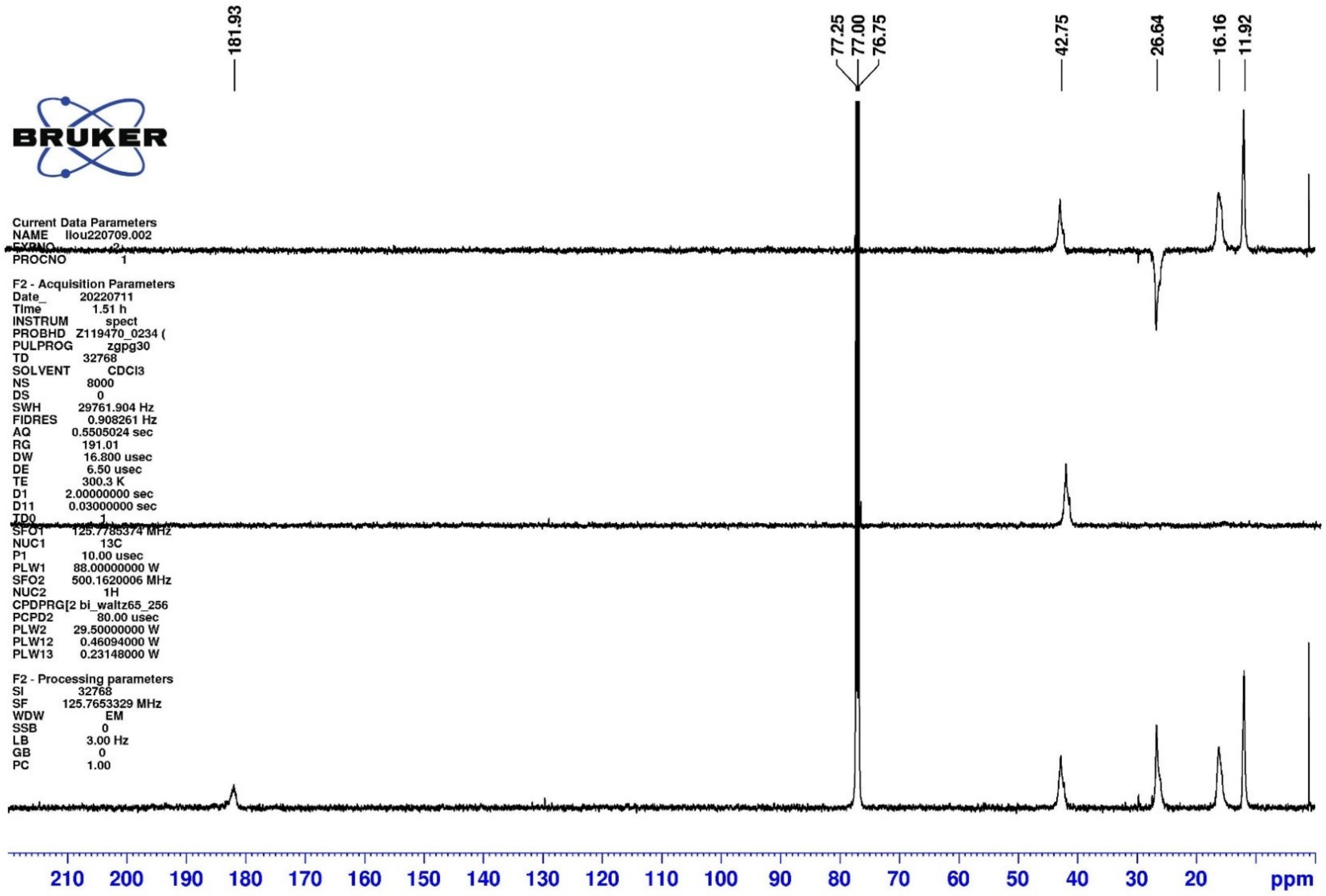

Figure S6. ^{13}C NMR of cluster **3**

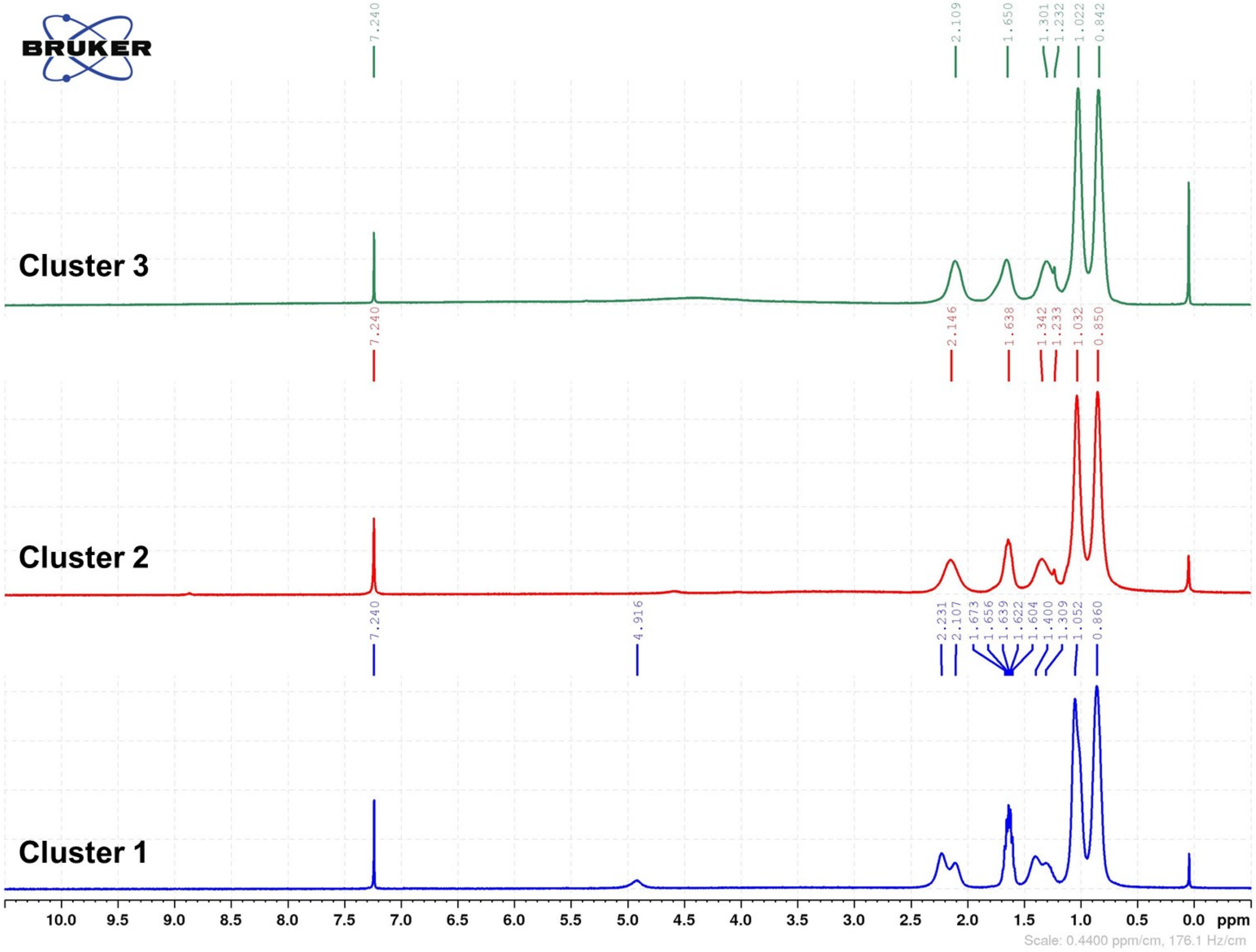

Figure S7. ^{1}H NMR of cluster **1**, **2** and **3**
